# Design and Synthesis of ^68^Ga‐Labeled Peptide‐Based Heterodimers for Dual Targeting of NTS_1_ and GRPR

**DOI:** 10.1002/cmdc.202400843

**Published:** 2025-02-16

**Authors:** Sacha Bodin, Santo Previti, Emmanuelle Jestin, Emmanuelle Rémond, Delphine Vimont, Frédéric Lamare, Imade Ait‐Arsa, Elif Hindié, Florine Cavelier, Clément Morgat

**Affiliations:** ^1^ University of Bordeaux CNRS EPHE INCIA UMR 5287 F-33400 Talence France; ^2^ CHU Bordeaux Department of Nuclear Medicine F-33000 Bordeaux France; ^3^ Pôle Chime Balard IBMM UMR 5247 CNRS Université Montpellier ENSCM F-34293 Montpellier France; ^4^ Department of Chemical, Biological, Pharmaceutical, and Environmental Sciences University of Messina Viale Stagno d'Alcontres 31 98166 Messina Italy; ^5^ GIP CYROI – Cyclotron Réunion Océan Indien F-97490 Saint Clotilde France; ^6^ Institut Universitaire de France IUF F-75000 Paris France

**Keywords:** Neurotensin, NTS_1_, Bombesin, GRPR, Heterodimers, PET imaging

## Abstract

Tumor heterogeneity remains one of the main obstacles for cancer diagnosis and treatment. The simultaneous targeting of several cancer biomarkers is an appealing approach for improved diagnostic procedures. Neurotensin receptor 1 (NTS_1_) and Gastrin‐Releasing Peptide Receptor (GRPR) are both G‐protein coupled receptors with complementary profile of expression in several cancer types. This work proposes the design, the synthesis and the *in vitro* radiopharmaceutical characterization of three heterodimers, based on GRP/NT modified peptides, radiolabeled with gallium‐68. Two NTS_1_/GRPR targeting pharmacophores containing linear hybrids that differ in the *C*‐terminus were synthesized (i. e., JMV 7110 and JMV 7253). The branched analogue of the silicon‐containing heterodimer JMV 7110, namely JMV 7266, was also synthesized. After radiolabeling with ^68^Ga, saturation binding studies performed on HT29 (NTS_1_^+^/GRPR^−^) and PC3 (NTS_1_^+^/GRPR^+^) cells demonstrated a significant loss in NTS_1_ and GRPR affinity compared to the reference monomers with the exception of the NTS_1_ affinity of [^68^Ga]Ga‐JMV 7266 which was preserved. Considering cellular processing, NTS_1_‐internalization at 1 h was the highest with [^68^Ga]Ga‐JMV 7266 and was similar to the reference compound. Interestingly [^68^Ga]Ga‐JMV 7266 demonstrated lower efflux than the other linear heterodimers but also than its NT reference compound. The branched structure of [^68^Ga]Ga‐JMV 7266 seems beneficial for dual NTS_1_/GRPR targeting.

## Graphical abstract

Tumor heterogeneity is challenging for cancer diagnosis and treatment. This study designs and evaluates three gallium‐68 radiolabeled heterodimers targeting two G‐protein coupled receptors, namely NTS1 (neurotensin receptor 1) and GRPR (gastrin‐releasing‐peptide receptor). The branched analogue [68Ga]Ga‐JMV 7266 preserves NTS1 affinity, exhibits higher internalization and reduced efflux, highlighting its potential as a promising dual‐targeting agent for improved cancer imaging.

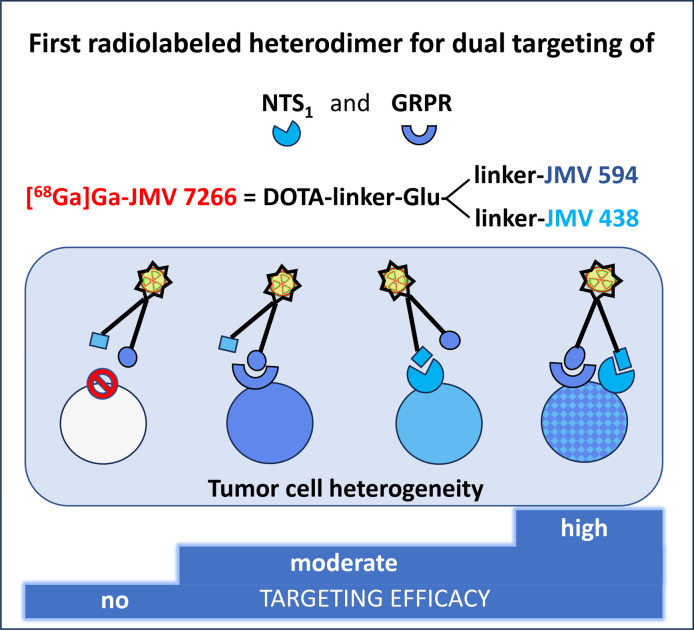

## 1 Introduction

Cancer markers can be differentially expressed in tumor cells either at different stages of the disease or at different times of the pathology, leading to spatial and/or temporal heterogeneity.[^1^, ^2^] Precision medicine aims at considering patient‐specific markers to provide personalized imaging/therapeutic procedures. Among the biomarkers of interest, neurotensin (NT) receptors and Gastrin Releasing Peptide (GRP) receptor, were found to be differentially overexpressed in a panel of cancers.[^3^, ^4^] In breast cancer NTS_1_ is overexpressed in about one third of primary breast cancers and in 73 % of associated metastatic lymph nodes from NTS_1_‐positive primaries.^[5]^ Regarding phenotypes, triple negative (ER‐, PR‐, HER2‐) tumors, express high amounts of NTS_1_. Moreover, NTS_1_ expression is correlated with the tumor size and the number of metastatic lymph nodes.^[6]^ Regarding outcomes, NTS_1_ overexpression is associated with shorter 10 year metastasis free interval.^[6]^ Contrarily, GRPR expression was found (86 %) in hormone dependent, low proliferating tumors (i. e. luminal A phenotype, positive to hormone receptors).[^7^, ^8^] The same opposite pattern of expression was found in colon cancer where NTS_1_ is overexpressed in 76 % of colon adenocarcinomas^[9]^ and is associated with inflammatory bowel disease‐related oncogenesis^[10]^ while it is undetectable in normal colonic epithelium. GRPR was associated with good prognosis factors and low metastasis risk.[^11^, ^12^] Finally, in prostate cancer NTS_1_ overexpression has been found in PSMA‐negative primary tumors^[13]^ and in metastatic lymph nodes^[14]^. In primary prostate cancer, low grade lesions are frequently GRPR‐rich.[^14^, ^15^, ^16^]

Overall, NTS_1_ and GRPR stand as two promising targets for solid tumors imaging and therapy considering their complementary profiles of expression. Therefore, the development of radiolabeled GRP/NT heterodimers makes sense to detect and image tumors with heterogeneous cell populations within the same tumor. In this study, we aimed at synthetizing three heterodimers based on GRP/NT modified peptide‐based sequences and derivatized with a chelator for radiolabeling with Gallium‐68 (^68^Ga). The resulting ^68^Ga‐labeled heterodimers were next fully characterized *in vitro* in colon cancer HT29 cells (NTS_1_^+^/GRPR^−^) and in prostate cancer PC3 cells (NTS_1_^+^/GRPR^+^) cells.

The design of novel heterodimers was based on SAR studies. Substituting Arg^8^–Arg^9^ in the pharmacophore NT[8–13] with Lys^8^–Lys^9^ did not alter the biological activity, while facilitating the synthesis of NT analogues.[^17^, ^18^] For this reason, the NT analogue JMV 438 ([Lys^8^–Lys^9^]NT[8–13]) was widely studied and validated as a positive control (NTS_1_ *K*_i_=4.0 nM, Chart 1).^[19]^ Recently, in the attempt to improve the binding affinity towards NTS_1_, lipophilic residues were incorporated in the NT[8–13] sequence.^[20]^ Among the new analogues, JMV 2007 (Chart 1), in which a trimethylsilylalanine (TMSAla) residue was inserted at the position 13 ([Lys^8^‐Lys^9^‐TMSAla^13^]NT[8–13]), showed an IC_50_ value towards NTS_1_ in the picomolar range (NTS_1_, IC_50_=0.02  nM). These findings highlighted the crucial role played by the free *C*‐terminus for a strong binding affinity towards the subtype 1 of NT receptors.[^21^, ^22^]


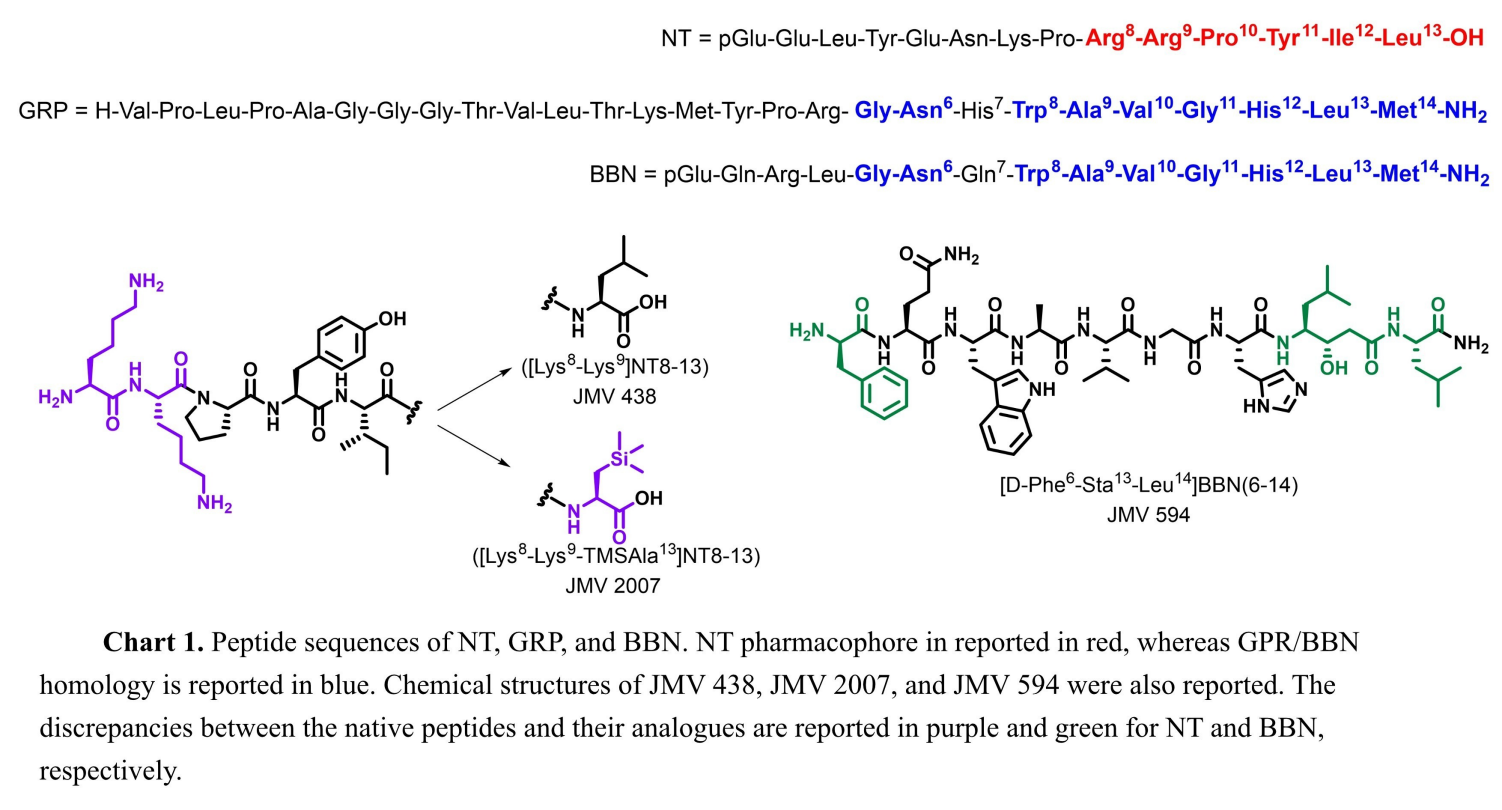


With regard to GRP, incorporation of statine (Sta) and nor‐statine (nor‐Sta) residues at the position 13 of truncated BBN analogues, along with additional modifications, such as D‐Phe^6^ and Leu^14^ led to the identification of potent GRPR antagonists mimicking the transition state, resulting in the failure of receptor activation.^[23]^ Among them, JMV 594 ([D‐Phe^6^,(3*S*,4*S*)Sta^13^‐Leu^14^]BBN(6–14)), was found to be the most interesting antagonist (Chart 1), paving the way towards the development of potent BBN analogues.[^24^, ^25^, ^26^] For all these reasons, the novel BBN/NT heterodimers herein reported carry JMV 594 as the only BBN pharmacophore, whereas JMV 438 and JMV 2007 were alternatively inserted as NT analogues in the linear heterodimers JMV 7110 and JMV 7253, respectively (Chart 2). Since the *C*‐terminal portion of NT analogues plays a key role in the binding to NTS_1_, the new compounds bear the NT pharmacophore at the *C*‐terminus. JMV 594 and JMV 438/JMV 2007 were anchored by two residues of βAla, that function as a linker between the two ligands. Lastly, DOTA was selected as chelator agent, and it was anchored to JMV 594 by two residues of βAla. Whilst Leu^14^ is the *C*‐terminus in JMV 594, in the linear heterodimers it is involved in a peptide bond with the linker. In order to address the discrepancy between the parent ligand and the new heterodimers, the branched analogue JMV 7266 was created, incorporating both JMV 438 and JMV 594 pharmacophores with a free *C*‐terminus (Chart 2). A residue of Glu was employed to link the ligands through the two −COOH groups, while DOTA was anchored to Glu through a βAla residue.


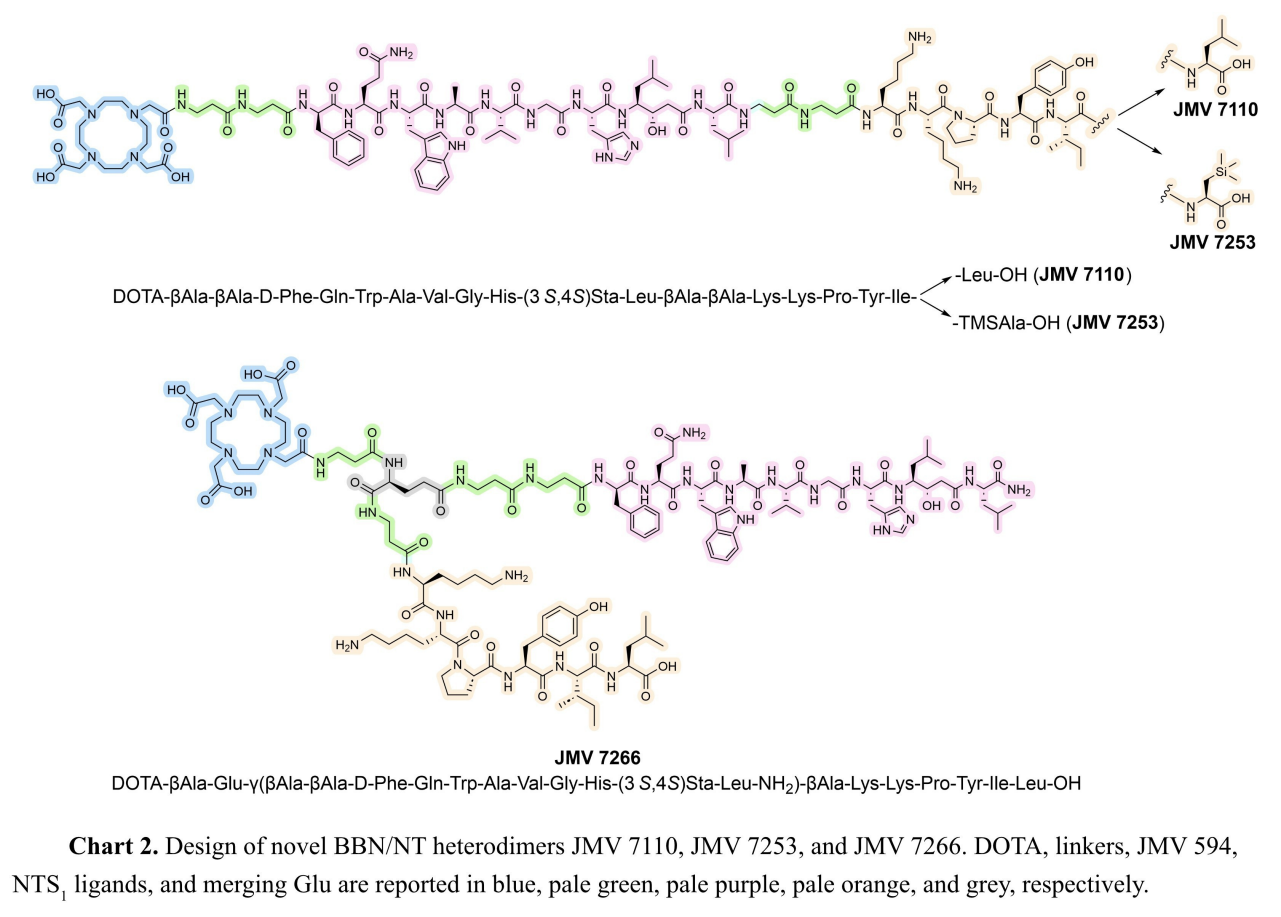


## 2 Results

### 2.1 Synthesis

The synthesis of the target linear heterodimers JMV 7110 and JMV 7253 was performed using Solid Phase Peptide Synthesis (SPPS), whereas the branched heterodimer JMV 7266 was obtained using a hybrid synthetic pathway, that combined SPPS with synthesis in solution, similar to that reported recently by us.^[27]^ JMV 7110 was synthesized starting from commercially available Fmoc‐Leu‐Wang resin with a loading of 0.7 mmol/g at a scale of 0.1 mmol. JMV 7253 was synthesized starting from Fmoc‐TMSAla‐2‐chloro trityl resin (2‐CTC) with a loading of 0.8 mmol/g at a scale of 0.1 mmol. The synthesis of Fmoc‐TMSAla‐OH was detailed in our earlier reports.[^28^, ^29^] For JMV 7266, synthesis commenced with Fmoc‐Leu Rink amide resin with a loading of 0.4208 mmol/g at a scale of 0.08 mmol. The NT portion NT11–13, namely H‐Tyr(O*t*Bu)‐Ile‐Leu‐O*t*Bu was synthesized in solution using Fmoc‐chemistry, whereas the remaining NT arm, Fmoc‐Glu(OMe)‐βAla‐Lys(Boc)‐Lys(Boc)‐Pro‐OH, was obtained starting from Fmoc‐Pro‐2CTC resin with a loading of 0.68 mmol/g. The three final compounds JMV 7110, JMV 7253, and JMV 7266 were obtained with yields of 6 %, 5 %, and 2 %, respectively. After purification, all the three heterodimers showed a purity greater than 98 %. Detailed chemical data can be found in the Supporting Information.

### 2.2 ^68^Ga Radiolabeling

Radiolabeling was performed at moderate yields of 44.4±6.3 % (mean value for all radiolabelings for the three compounds, after purification, not decay‐corrected). Specific activities (at end‐of‐synthesis) were respectively 15±7 MBq/nmol for [^68^Ga]Ga‐JMV 7110, 20±7 MBq/nmol for [^68^Ga]Ga‐JMV 7253 and 21±10 MBq/nmol for [^68^Ga]Ga‐JMV 7266. All compounds were obtained at radiochemical purities >95 %. Representative radio‐HPLC chromatograms are displayed in the Supporting Information.

### 2.3 Lipophilicity

The linear heterodimer [^68^Ga]Ga‐JMV 7110 bearing the JMV 594 and JMV 438 sequences has a LogD_7.4_ value of −2.74±0.21. The branched heterodimer [^68^Ga]Ga‐JMV 7266 bearing the same binding sequences exhibit a similar LogD_7.4_ value of −2.62±0.13. Finally, the linear heterodimer [^68^Ga]Ga‐JMV 7253 bearing JMV 594 and JMV 2007 sequences has a slightly lower hydrophilicity value of −2.46±0.07 that can be attributed to the TMSAla as already reported.^[28]^

### 2.4 In Vitro Studies

#### 2.4.1 Affinity Studies

**Figure 1:**
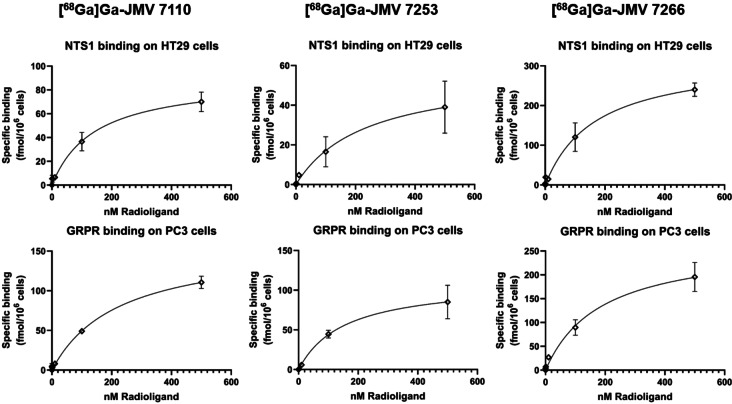
Saturation binding curves of the ^68^Ga‐heterodimers performed on HT29 and PC3 cells.

K_D_ values of the linear heterodimer [^68^Ga]Ga‐JMV 7110 were 173±41 nM at NTS_1_ (Bmax=177±123 fmol/10^6^ cells) and 165±84 nM at GRPR (Bmax=227±96 fmol/10^6^ cells). [^68^Ga]Ga‐JMV 7266, having the same binding sequences but in a branched structure, showed similar affinity values of 165±15 nM at NTS_1_ (Bmax=258±92 fmol/10^6^ cells) and 142±39 nM at GRPR (Bmax=232±49 fmol/10^6^ cells). The linear heterodimer, [^68^Ga]Ga‐JMV 7253 showed similar affinity than the two other heterodimers of 188±66 nM at NTS_1_ (Bmax=50±10 fmol/10^6^ cells) and 171±23 nM at GRPR (Bmax=106±7 fmol/10^6^ cells) (Figure 1).

#### 2.4.2 Cell Processing Studies

**Figure 2:**
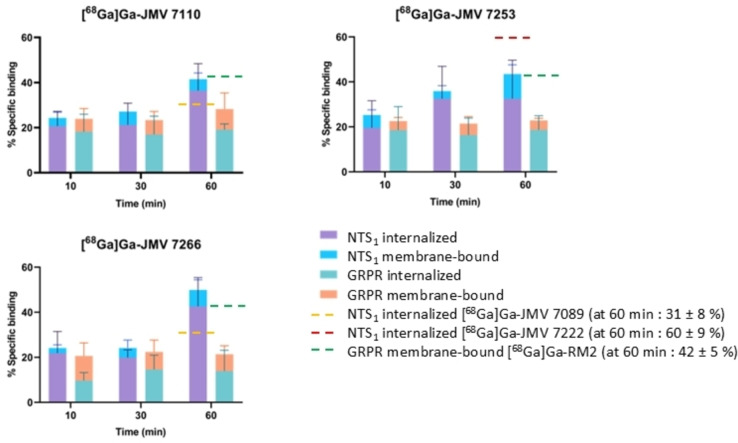
Intracellular distribution of the newly developed ^68^Ga‐heterodimers on HT29 and PC3 cells. Values from reference compounds are reported in dashed lines at 60 min only for easier reading.

Kinetic studies indicate that the NTS_1_‐mediated internalized fraction rapidly reached 20 % 10 minutes after incubation and increased up to 36±12 %, 33±17 %, and 43±13 % on HT29 cells at 60 minutes for [^68^Ga]Ga‐JMV 7110, [^68^Ga]Ga‐JMV 7253, and [^68^Ga]Ga‐JMV 7266, respectively (Figure 2). GRPR‐mediated internalization assessed on PC3 cells rapidly reached its maximum value (10 min) and remained stable until 60 minutes. GRPR‐internalized fraction were 19±3 %, 19±6 %, and 14±9 % for [^68^Ga]Ga‐JMV 7110, [^68^Ga]Ga‐JMV 7253, and [^68^Ga]Ga‐JMV 7266, respectively. The NTS_1_‐membrane‐bound fraction was always below 8 % of total binding, excepted for [^68^Ga]Ga‐JMV 7253 (11±4 % at 60 min). The GRPR‐mediated membrane‐bound fraction was always below 10 % of total binding at 60 minutes for all radioprobes.

### 2.5 Efflux Studies

**Figure 3:**
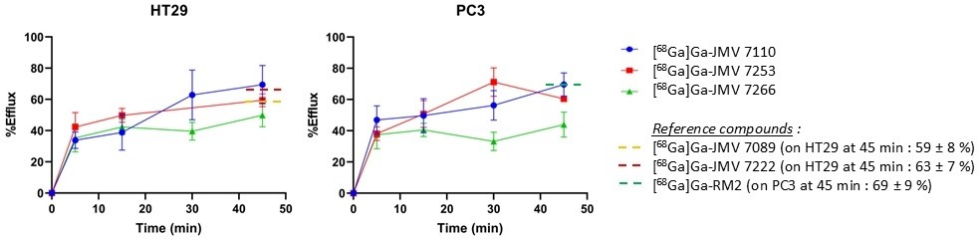
Efflux of [^68^Ga]Ga‐heterodimers on HT29 and PC3 cells. Values from reference compounds are reported in dashed lines at 45 min only for easier reading.

Externalization assays were carried out on HT29 and PC3 cells (Figure 3). [^68^Ga]Ga‐JMV 7110 and [^68^Ga]Ga‐JMV 7253 demonstrated moderate efflux five minutes post incubation (34±5 % and 42±9 % respectively on HT29 cells and 47±9 % and 38±4 on PC3 cells). These values increased up to 69±12 % and 59±4 % on HT29 and 70±7 % and 60±1 % on PC3 cells at 45 min. Interestingly, [^68^Ga]Ga‐JMV 7266 was less externalized with efflux values of 35±9 % on HT29 and 37±9 % on PC3 at 5 min and 50±7 % on HT29 and 44±8 % on PC3 at 45 min.

Overall, [^68^Ga]Ga‐JMV 7266 displayed affinity towards NTS_1_ and GRPR in the upper nanomolar range, receptor‐mediated time‐dependent internalization and importantly lower efflux in this series of heterodimers.

## 3 Discussion

Among the variety of tumour surface markers, targeting neuropeptide receptors has been a successful approach for nuclear imaging and therapy of patients suffering from neuroendocrine tumours.^[30]^ Other neuropeptide receptors are on the horizon.^[31]^ Among them, the Gastrin‐Releasing Peptide Receptor (GRPR) and the neurotensin receptor‐1 (NTS_1_) hold great promise. The emergence of heterodimeric peptides as novel targeting ligands for tumour imaging and therapy is supported by their potential enhanced binding affinity, improved specificity and improved pharmacokinetic properties.^[32]^ Therefore, in this work we were interested in developing a small series of radiolabeled heterodimers for dual targeting of NTS_1_ and GRPR.

**Scheme 1:**
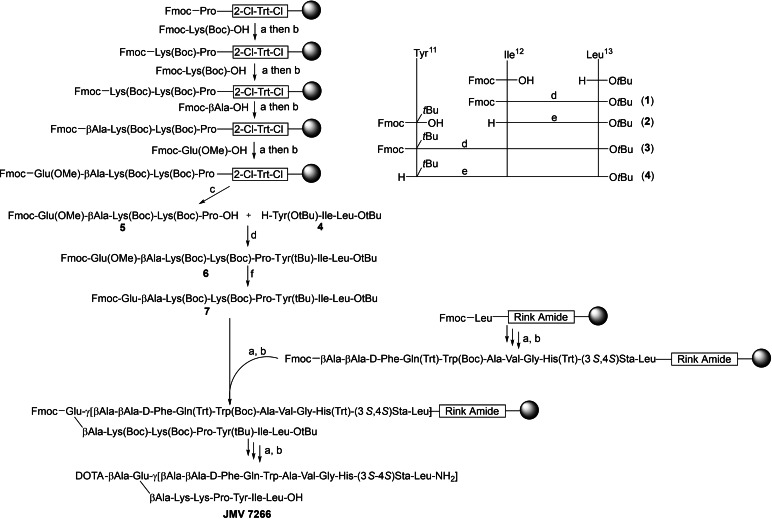
Reagents and conditions: a) 20 % piperidine in DMF (5 min×3), rt; b) Appropriate amino acid, HATU, DIPEA, DMF, appropriate time/cycle, rt; c) DCM/TFE/AcOH 8 : 1 : 1, 12 h, rt; d) HATU, DIPEA, DMF, rt, on; e) 20 % piperidine in DMF, rt, 1 h; f) NaOH, CaCl_2_, *i*PrOH/H_2_O (7 : 3), 7 h, rt. All reactions and conditions are detailed reported in the Supporting Information.

JMV 7110 and JMV 7253 were synthesized following the same synthetic approach, described in the experimental section and Supporting Information, with the only difference being the incorporation of the unnatural amino acid TMSAla, which was synthesized and loaded on 2‐Cl‐Trt chloride resin as we previously reported.[^28^, ^33^] The branched heterodimer JMV 7266 was synthesized using a hybrid approach combining SPPS and solution synthesis, based on a method we recently reported.^[27]^ This strategy was necessary since the two pharmacophores had different C‐terminal functional groups – one as a carboxylic acid and the other as an amide – requiring distinct synthesis methods. Considering that JMV 594 harbors an amide group at the *C*‐terminus and expensive amino acid at the position 13 (Sta), the GRPR ligand was synthesized through SPPS starting from Fmoc‐Leu‐Rink amide resin with a loading of 0.4208 mmol/g (Scheme 1). The two residues of βAla were also anchored by SPPS. On the other hand, NT ligand was obtained by a hybrid strategy, involving both SPPS and synthesis in solution. This approach aimed to obtain the NT arm chemically suitable to be anchored to the GRPR ligand through the SPPS. In order to be properly added to the spacer of JMV 594, the NT arm should have *i*) a carboxylic group (such as the γ‐COOH of Glu), *ii*) piperidine‐labile protecting groups at the *N*‐terminus, and *iii*) acid‐labile protecting groups for −OH, −NH_2_, and −COOH. Additionally, the γ‐COOH of Glu had to be protected with a substituent that could be easily removable while preserving Fmoc, Boc, and O‐*t*Bu protecting groups.

In light of this, we decided to split the NT arm in two fragments, namely the NT[11–13] portion H‐Tyr(*t*Bu)‐Ile‐Leu‐O*t*Bu (**4**) and the remaining pentapeptide Fmoc‐Glu(OMe)‐βAla‐Lys(Boc)‐Lys(Boc)‐Pro‐OH (**5**). The Glu residue was incorporated as a methyl ester at the γ‐COOH, which is stable under piperidine and acid conditions but can be converted to a carboxylic group without affecting the Fmoc and acid‐labile protecting groups. The tripeptide H‐Tyr(*t*Bu)‐Ile‐Leu‐O*t*Bu **4** was synthesized in solution using Fmoc‐chemistry, while the remaining NT arm was obtained by SPPS using 2‐Cl‐Trt chloride resin, as shown in Scheme 1. In order to preserve the acid‐labile protecting groups, the final cleavage was performed using a mixture of DCM/TFE/AcOH (8 : 1 : 1). The subsequent coupling between the Fmoc‐Glu(OMe)‐βAla‐Lys(Boc)‐Lys(Boc)‐Pro‐OH and H‐Tyr(*t*Bu)‐Ile‐Leu‐O*t*Bu yielded intermediate **6**. The presence of Pro at the C‐terminus of the pentapeptide **5** helped prevent epimerization, which often occurs in coupling reactions when *N*‐unprotected amino acids are employed. Subsequently, the methyl ester of Glu was successfully hydrolyzed with NaOH in the presence of CaCl_2,_ in order to preserve the Fmoc group. The resulting intermediate **7** was then anchored to the JMV 594 ligand by SPPS. Following Fmoc‐deprotections/coupling reactions, we successfully synthesized the branched heterodimer JMV 7266.

This hybrid synthetic approach proved to be valid, feasible, and effective, with potential for further improvements to enhance yields.

The constructs were then derivatized with DOTA chelator for radiolabeling with the positron‐emitter ^68^Ga.

After radiolabeling, physicochemical characterization indicates that [^68^Ga]Ga‐JMV 7253 was significantly more lipophilic than [^68^Ga]Ga‐JMV 1110 and [^68^Ga]Ga‐JMV 7266 in line with the presence of the lipophilic amino acid TMSAla. Similar data have been obtained with radiolabeled NTS_1_‐agonists.^[28]^

The *in vitro* behaviors of the radiolabeled compounds were then investigated on colon cancer HT29 cells and prostate cancer PC3 cells and compared to the reference monomers to understand the consequences of heterodimerization on the biological activity. The monomers were JMV 7089 (DOTA‐βAla‐βAla‐**JMV 438)** regarding the NT pharmacophore used in JMV 7110 and JMV 7266, JMV 7222 (DOTA‐βAla‐βAla‐**JMV 2007)** for the NT pharmacophore in JMV 7253 and RM2 (DOTA‐JMV 594) for the BBN pharmacophore used in all heterodimers.

Saturation studies showed that very similar affinity values were obtained at the NTS_1_ and at the GRPR (range 140–190 nM) for [^68^Ga]Ga‐JMV 7110, [^68^Ga]Ga‐JMV 7266 and. Regarding [^68^Ga]Ga‐JMV 7253, the affinity values reported are largely decreased at both NTS_1_ and GRPR compared with the corresponding monomers. [^68^Ga]Ga‐JMV 7222 and [^68^Ga]Ga‐RM2 have affinity values of 4.3±0.5 nM towards the NTS_1_ and 0.25±0.19 nM towards the GRPR respectively. The cases of [^68^Ga]Ga‐JMV 7110 and [^68^Ga]Ga‐JMV 7266 are different as heterodimerization of the NT pharmacophore with JMV 594 does not promote a loss of NTS_1_‐affinity compared to the monomer [^68^Ga]Ga‐JMV 7089 (Kd (NTS_1_)=154.7±13.2 nM).[^27^, ^28^, ^34^] Contrarily, adding the NT pharmacophore to the BBN moiety yields a decreased in affinity towards GRPR>500.

Considering cellular processing, NTS_1_‐internalization at 1 h was the highest with [^68^Ga]Ga‐JMV 7266 which was similar to the reference [^68^Ga]Ga‐JMV 7089 (31±8 % of total cell‐radioactivity internalized at 60 min). A dramatic loss of NTS_1_‐specific internalization was depicted for [^68^Ga]Ga‐JMV 7253 compared to its monomer for the NT pharmacophore as expected ([^68^Ga]Ga‐JMV 7222 showed NTS_1_ specific internalization higher than 55 % of cell associated radioactivity). At the GRPR, the specific internalization reached 15–20 % which is very similar to [^68^Ga]Ga‐RM2.^[34]^ Unfortunately, the membrane‐bound fraction, which is characteristic to most antagonists, was lost as a consequence of the loss of affinity and/or a switch from antagonist to agonist occurred as already reported.^[35]^ Finally, [^68^Ga]Ga‐JMV 7266 was more trapped into the cells as evidenced by its weaker efflux compared to the reference [^68^Ga]Ga‐JMV 7089 (≈60 % at 45 min). Thus, the JMV 7266 structure paves the way for the development of optimized compounds to make in vivo uptake more likely. Given the affinity loss observed, no in vivo study no performed. Nevertheless, the in vitro data from this study are essential for future structural optimization of JMV 7266.

## 4 Conclusions

Overall, in this series of radiolabeled NT/BBN heterodimers, the branched structure of JMV 7266 is advantageous for targeting and tolerates well the BBN pharmacophore regarding NTS_1_ affinity, but this was not applicable to the NT pharmacophore. Further optimization is needed to improve affinity and targeting possibilities of BBN/NT heterodimers.

## Experimental Section

### Chemistry

Solvents and reagents employed for the synthesis of heterodimers and related intermediates were purchased from Merck. All *N*‐Fmoc‐protected natural amino acids and Fmoc‐βAla‐OH were obtained from Fluorochem, whereas Fmoc‐(3*S*,4*S*)Sta‐OH and Fmoc‐D‐Phe‐OH were purchased from IRIS Biotech. DOTA(O*t*Bu)_3_‐OH was obtained from TCI. Fmoc‐TMSAla‐OH was synthesized as we previously reported.^[29]^ For the synthesis in batch, all reactions involving air sensitive reagents were carried‐out in nitrogen atmosphere. A VWR Microplate Shaker was employed for the SPPS. 2‐Chloro‐Trityl chloride (2‐CTC) resin was purchased from Merck, whereas Fmoc‐Leu‐Wang, Fmoc‐Pro‐2‐CTC, and Fmoc‐Leu Rink Amide resins were obtained from IRIS Biotech. Purifications of the intermediates obtained in batch were carried‐out by column chromatography using silica gel, which was purchased from Merck (Merck 60, 230–400 mesh). Silica gel 60 F_254_ plates (obtained from Merck) were used for TLC. When target compounds were not UV‐visible, the TLC was treated with an ethanol solution of phosphomolybdic acid hydrate (15 %). The three final heterodimers were purified using a Gilson PLC 2250 preparative apparatus equipped with a C18 Deltapak column (100 mm×40 mm, 15 μM, 100 Å), with a flow rate equal to 20 mL/min. A mixture of water‐ and ACN‐containing 0.1 % TFA was employed, starting from 100 : 0 %–0 : 100 % of water and ACN, respectively. The gradient time was properly modified according to the run time of the target compounds. All the HPLC analysis were performed using a C18 Chromolith Flash 25×4.6 mm column, operating with a flow rate of 3 mL/min. Running HPLC was performed in gradient using water‐ and ACN‐containing 0.1 % of TFA as the solvents: starting from 100 % of water (solvent A), the % of ACN (solvent B) was increased to 100 % over 3 min. Characterization of all compounds was performed by LC/MS, which consists of a Water Alliance 2629 HPLC coupled with a ZQ spectrometer fitted with an electrospray source operating in positive ionization mode (ESI^+^). All data regarding the LC/MS system were already reported. High‐resolution mass spectra (HRMS) were performed by “Laboratoire de Mesures Physiques” of University of Montpellier (FR) as we previously reported.^[33]^

JMV 7110 was synthesized using SPPS from commercially available Fmoc‐Leu Wang resin with a loading of 0.7 mmol/g. The resin was first swollen in a mixture of DCM/DMF (4 : 1) for 20 min. The subsequent Fmoc‐deprotection/coupling reactions followed by final cleavage led to the expected compound, with a 6 % yield. Typically, coupling reactions were shaken for 45 min. However, given the presence of bulky and costly amino acids, some coupling reactions were repeated or prolonged. The same procedure was followed for the synthesis of JMV 7253, starting from Fmoc‐TMSAla 2‐CTC resin. The branched analogue JMV 7266 was obtained following the hybrid approach described above. Detailed synthesis data are provided in the Supplementary Information

### Radiolabeling Procedure

Radiolabeling was achieved using the FastLab automate cassette system (GE Healthcare). 1.1 ml of ^68^GaCl_3_ (≈200–400 MBq, GalliAd ^68^Ge/^68^Ga generator, IRE‐EliT, Belgium) with 50 μg of JMV 7110, JMV 7253, or JMV 7266, in acetate buffer 0.1 M (pH=4.6) and heated using microwave at 90 °C for 5 minutes. The crude peptide was then purified by a Sep‐Pak Light C_18_ cartridge (WAT023501) using 0.5 mL of ethanol and then formulated in PBS in order to obtain a final volume of 3 mL. Radiochemical purity was monitored with UV‐radio HPLC (Luna 4 mL/min, λ=220 nm C_18_; 150 mm×4,6 mm×5 μm) and TLC analysis using methanol/ammonium acetate 1 M (1 : 1). HPLC conditions were as follow: 0–2 min: 90 % water with 0.1 %TFA (A), 10 % ACN (B), 2–10 min: from 90 %–10 % A; 10–12 min: 10 % A; 12–14 min: from 10 %–90 % A.

### Determination of the Octanol/Water Partition Coefficient (LogD_7.4_)

LogD determination was performed in triplicate and repeated over three radiolabelings. Approximately 3.7 MBq of the ^68^Ga‐radiolabeled peptide were added to qsp 500 μL of PBS (Gibco, France, pH=7.4) and 500 μL of octanol (Sigma Aldrich, USA) in microcentrifuge tubes. Tubes were shaken then vortexed and centrifuged at room temperature (3 min, 4000 rpm). 100 μL of each layer were sampled in separate test tubes and measured in a gamma‐counter (Wizard2, PerkinElmer, USA). LogD_7.4_ value was obtained by calculating the log_10_ of the ratio between the organic phase activity (cpm) and the aqueous phase activity (cpm). Both activities were decay‐corrected.

### Cell Culture

Cells were obtained from the University of Bordeaux and no additional authentication was performed. The human colorectal adenocarcinoma HT29 cells were cultured at 37 °C and 5 % CO_2_ in RPMI medium (Gibco, France) supplemented with 400 UI/mL penicillin and 400 μg/mL streptomycin (PenStrep, Gibco, France), 10 % (v/v) of FBS and 1 % (v/v) of L‐alanine‐L‐glutamine (GlutaMAX, Gibco, France). The human prostate cancer PC3 cells were cultured at 37 °C and 5 % CO_2_ in DMEM/F12 medium (Gibco, France) supplemented with 400 UI/mL penicillin and 400 μg/mL streptomycin (PenStrep, Gibco, France), 10 % (v/v) of FBS and 1 % (v/v) of L‐alanine‐L‐glutamine (GlutaMAX, Gibco, France).

### In Vitro Studies

HT29 and PC3 cells were seeded into 24 wells plates at the density of 2.10^5^ cells per well, the day before the experiment and incubated overnight at 37 °C and 5 % CO_2_. Binding, internalization and efflux assays were carried out at least three times in quadruplicate. HT29 cells were used to characterize neurotensin receptor binding (as described previously^[33]^) and PC3 cells were used to study bombesin receptor binding. For receptor‐mediated experiments, to avoid potential interference with other receptor subtypes, three blocking agents were used simultaneously. For example, when GRPR binding was studied, NMBR, NTS_1_ and NTS_2_ were blocked simultaneously using specific ligands. The following blocking agents were used at 1 μM concentration: neurotensin to block NTS_1_, levocabastin to block NTS_2_, bombesin to block GRPR and neuromedin B to block NMBR. Saturation binding studies, cell processing studies regarding internalized and membrane‐bound fractions, and efflux studies were performed. Detailed methodologies are described in supplemental information.

### Saturation Binding Studies

Plates were stored at 4 °C for 30 minutes in order to reduce cell processing and then incubated for 2 hours at 37 °C with 250 μL complete medium containing the radiolabeled peptide at increasing concentrations (0.1, 1, 10, 100, 500 nM) with and without saturation ligands. After incubation, the cells were rinsed twice with ice‐cold DPBS (250 μL, Gibco). Then the cells were lysed with NaOH 1 M (750 μL) to collect the bound fraction. Radioactivity of each fraction was determined in a gamma‐counter (Wizard2, PerkinElmer, USA). Affinity (K_D_) was determined by non‐linear regression using Prism 9.0 software (GraphPad Software Inc., USA). Experiments were performed three times in quadruplicates.

### Cellular Processing

1 MBq of ^68^Ga‐radiolabeled heterodimer or monomer in 250 μL complete medium with and without 1 μM of blocking ligands were added in each well. Plates were incubated for 10, 30 or 60 minutes at 37 °C. Three minutes before the selected time point, plates were stored at 4 °C to stop the internalization process. Media were removed and wells were rinsed three times with ice‐cold DPBS (250 μL). 250 μL of sodium acetate (20 mM, pH 5) were added in each well twice and then collected in tubes after 5 min incubation. Then the cells were lysed with NaOH 1 M (750 μL) to collect the bound fraction. Radioactivity of each fraction was determined in a gamma‐counter. Cell‐associated radioactivity results are expressed as percentage of specific binding.

### Efflux Studies

1 MBq of ^68^Ga radiolabeled heterodimers in 250 μL complete medium were added in each well. Plates were incubated 30 minutes at 37 °C. Media were removed and the cells were rinsed with ice‐cold DPBS (250 μL). 250 μL of sodium acetate (20 mM, pH 5) were added in each well and removed after 5 minutes incubation. The cells were rinsed again with ice‐cold DPBS (250 μL). Then 250 μL medium were added in each well and plates were incubated for 5, 15, 30 or 45 min at 37 °C. Media were collected in tubes and each well was rinsed twice with ice‐cold DPBS (250 μL). Then the cells were lysed with NaOH 1 M (750 μL) to collect the bound fraction. Radioactivity of each fraction was determined in a gamma‐counter. Results are expressed as percentage of total binding.

### Statistical Analyses

Statistical analyses were performed using Prism v9 software (GraphPad Software Inc., USA). Unpaired data were used. Quantitative values were expressed as means±SD. Statistical analysis with at least three groups were performed with the Kruskal‐Wallis test corrected for multiple comparisons using the Dunn's method. Comparisons between two groups were performed using the bilateral non‐parametric Mann‐Whitney test. P‐value<0.05 was considered significant.

## 5 Supporting Information Summary

The Supporting Information includes synthesis and characterization of intermediates and final compounds as well as radio‐HPLC profiles of the ^68^Ga‐heterodimers.

## 6 Abbreviations

NT: neurotensin
NTS_1_
: neurotensin receptor subtype 1
NTS_2_
: neurotensin receptor subtype 2
GRPR: gastrin‐releasing peptide receptor
NMBR: neuromedin B receptor
PET: positron emission tomography
PIN: prostatic intraepithelial neoplasia
ISUP: international society of urological pathology

## 7 Funding

This project was funded by INCa (project THERACAN).

## Conflict of Interests

The authors declare no conflict of interest.

8

## Supporting information

As a service to our authors and readers, this journal provides supporting information supplied by the authors. Such materials are peer reviewed and may be re‐organized for online delivery, but are not copy‐edited or typeset. Technical support issues arising from supporting information (other than missing files) should be addressed to the authors.

Supporting Information

## Data Availability

The data that support the findings of this study are available from the corresponding author upon reasonable request.
