# Supplementary material for: Design and Synthesis of 68Ga‐Labeled Peptide‐Based Heterodimers for Dual Targeting of NTS1 and GRPR
Source: ChemMedChem. 2025 Feb 16;20(9):e202400843. doi: 10.1002/cmdc.202400843 (PMC12058244; doi:10.1002/cmdc.202400843)
Supplement: Supplementary file 1 — Supporting Information [file CMDC-20-e202400843-s001.pdf]

ChemMedChem

Supporting Information

Design and Synthesis of ^{68}Ga-Labeled Peptide-Based Heterodimers for Dual Targeting of NTS_1 and GRPR

Sacha Bodin, Santo Previti, Emmanuelle Jestin, Emmanuelle Rémond, Delphine Vimont, Frédéric Lamare, Imade Ait-Arsa, Elif Hindié, Florine Cavelier,* and Clément Morgat

SUPPLEMENTARY INFORMATION

Design and synthesis of ^{68}Ga-labeled peptide-based heterodimers for dual targeting of NTS_1 and GRPR

Sacha Bodin[a,b,#], Santo Previti[c,d,#], Emmanuelle Jestin[e], Emmanuelle Rémond[c], Delphine Vimont[a], Frédéric Lamare[a,b], Imade Ait-Arsa[e], Elif Hindié[a,b,f], Florine Cavelier[c,*], Clément Morgat[a,b]

[a] *University of Bordeaux, CNRS, EPHE, INCIA UMR 5287, F-33400 Talence, France.*
[b] *CHU Bordeaux, Department of Nuclear Medicine, F-33000 Bordeaux, France.*
[c] *Pôle Chime Balard, IBMM, UMR 5247, F-34293 Montpellier, France.*
[d] *Department of Chemical, Biological, Pharmaceutical, and Environmental Sciences, University of Messina, Viale Stagno d'Alcontres 31, 98166 Messina, Italy.*
[e] *GIP CYROI – Cyclotron Réunion Océan Indien, F-97490 Saint Clotilde, France.*
[f] *Institut Universitaire de France, IUF, F-75000, Paris, France.*

[#]These authors contributed equally.

Corresponding author: *Pôle Chime Balard, IBMM, UMR 5247, CNRS, Université Montpellier, ENSCM,

F-34293 Montpellier, France. orcid.org/0000-0001-5308-6416; email: florine.cavelier@umontpellier.fr

Index

1. Synthesis of linear heterodimer JMV 7110 3

2. Characterization of JMV 7110.. 5

3. Synthesis of linear heterodimer JMV 7253 8

4. Characterization of JMV 7253.. 9

5. Synthesis of branched heterodimer JMV 7266 and its intermediates 13

6. Characterization of JMV 7266.. 18

7. Radiolabeling of heterodimer compounds.. 21

a. [^{68}Ga]Ga-JMV 7110 21

b. [^{68}Ga]Ga-JMV 7253 21

c. [^{68}Ga]Ga-JMV 7266.. 22

1. Synthesis of linear heterodimer JMV 7110

JMV 7110 was synthesized using SPPS starting from Wang resin preloaded with Fmoc-Leu (0.7 mmol/g) in a scale of 0.1 mmol (Scheme S1). A 10 mL plastic syringe equipped with a teflon filter was employed. Solvents inside the syringe was sucked off by filtration. The resin was swollen in a mixture of DCM/DMF (4 mL, 4:1) for 20 min. Fmoc deprotection was performed using a solution of 20% piperidine in DMF (4 mL, 3 x 5 min), and the resin was then washed with DCM (8 mL, x 3). Subsequently, DMF (4 mL) was added to the syringe, followed by the appropriate Fmoc-amino acid (4 eq.), HATU (4 eq.), and DIPEA (5 eq.). The syringe was shaken for 45 min or for different time, according to the side chain steric hindrance of the amino acids (see Scheme S1). Coupling reactions involving constrained or bulky amino acids and DOTA(*t*Bu)$_3$-OH were performed in duplicate using the same conditions. After this time, the coupling solution was sucked off and the resin was washed with DCM (4 mL, x 3) and DMF (4 mL x 1). The final cleavage was carried-out using a mixture of TFA/DCM/TIS (90:5:5, 4 mL) for 12 h. Subsequently, the solution was filtered in cold diethyl ether and centrifugated. Supernatant was carefully removed and the final product was purified by preparative HPLC. Yield 6%. Purity 99%. ESI-MS (t_R = 1.21 min): 843.7 [(M+3H$^+$)/3], 633.0 [(M+4H$^+$)/4], 506.6 [(M+5H$^+$)/5]. HPLC t_R 2.12 min; HRMS for $C_{121}H_{189}N_{29}O_{30}$ = calcd.: 1264.2069 [(M+2H$^+$)/2]; found: 1264.2072.

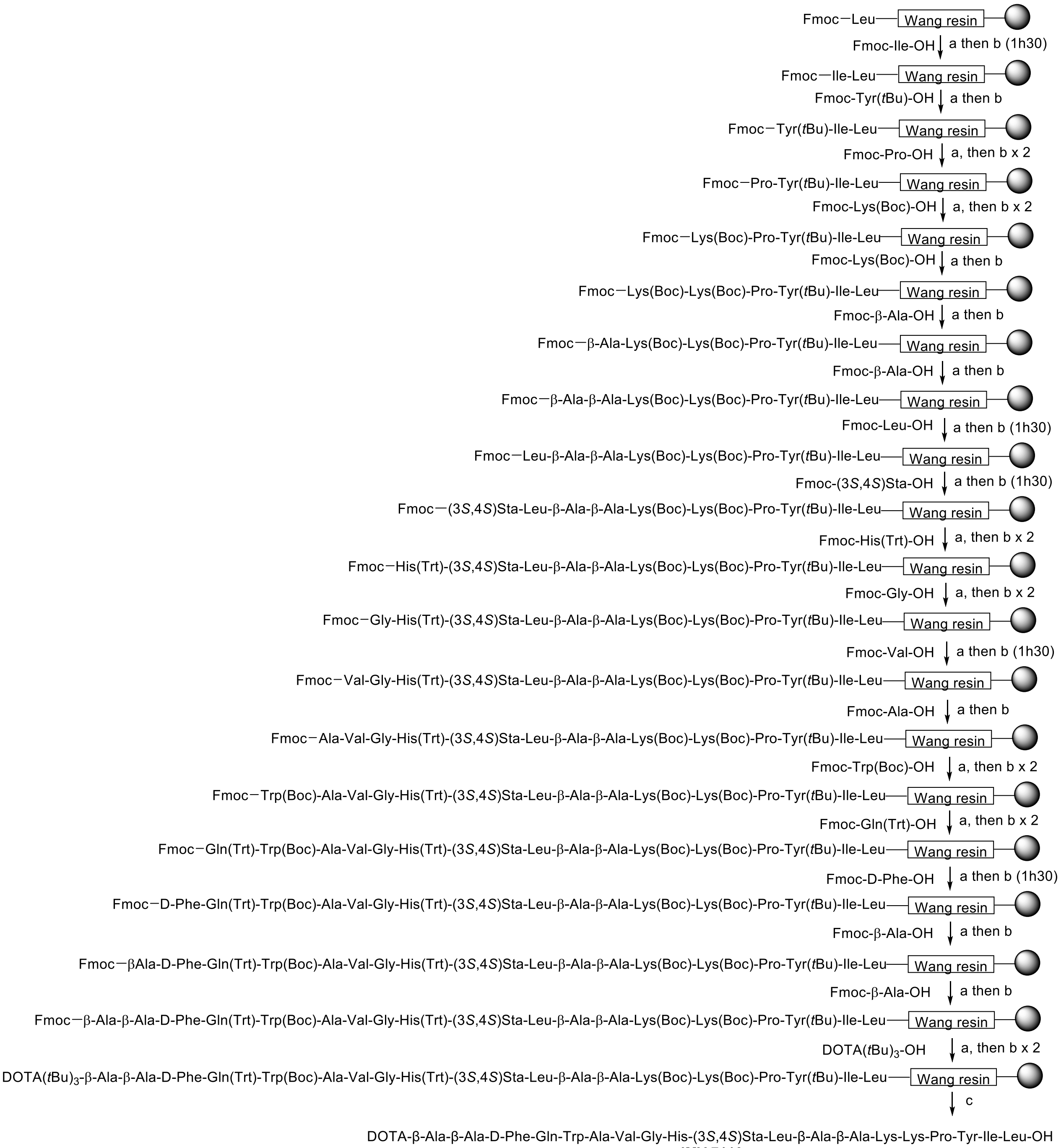

JMV 7110

Figure S1. Reagents and conditions: a) 20% piperidine in DMF (5 min x 3), rt; b) Appropriate amino acid, HATU, DIPEA, DMF, 45 min, rt; c) TFA/DCM/TIS 90:5:5, 12h, rt. The shaking time and "x 2" reported in brackets refer exclusively to conditions "b".

2. Characterization of JMV 7110

**Sample name:**	SPR082_prep_29
**Data file:**	C:\Chem32\LC1220_000\Agilent1220_000\Data\SantoPreviti\SPR082_prep_29 2018-10-12 10-35-15.D
**Description:**	
**Injection volume:**	5.000
**Instrument:**	LC 1220
**Injection date:**	10/12/2018 10:54:56 AM
**Acq. method:**	0a100_3min_214nm.M

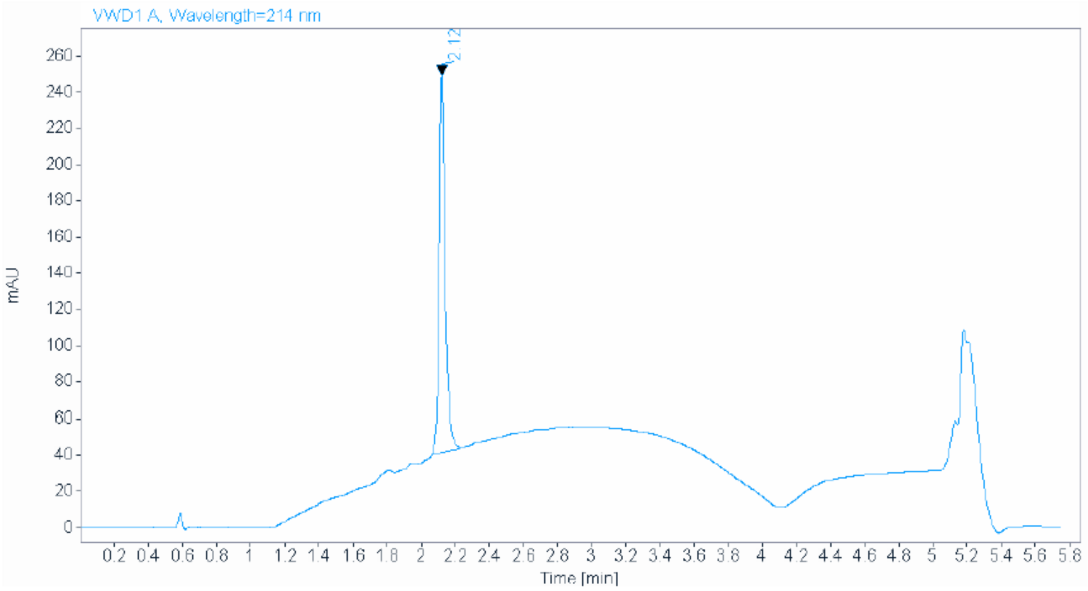

Signal: VWD1 A, Wavelength=214 nm

RT [min]	Area	Height	Peak Area Percent
2.120	492.34833	208.80443	100.00

Figure S2. HPLC profile of JMV 7110.

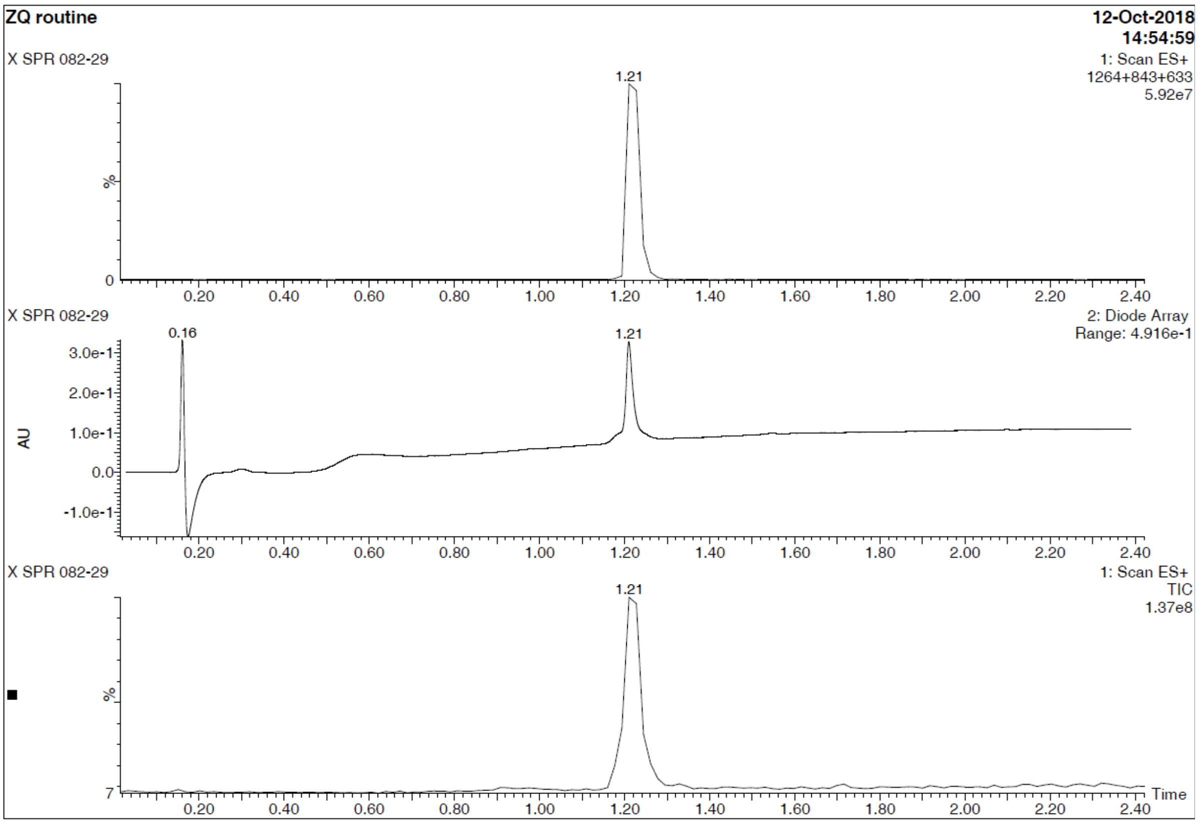

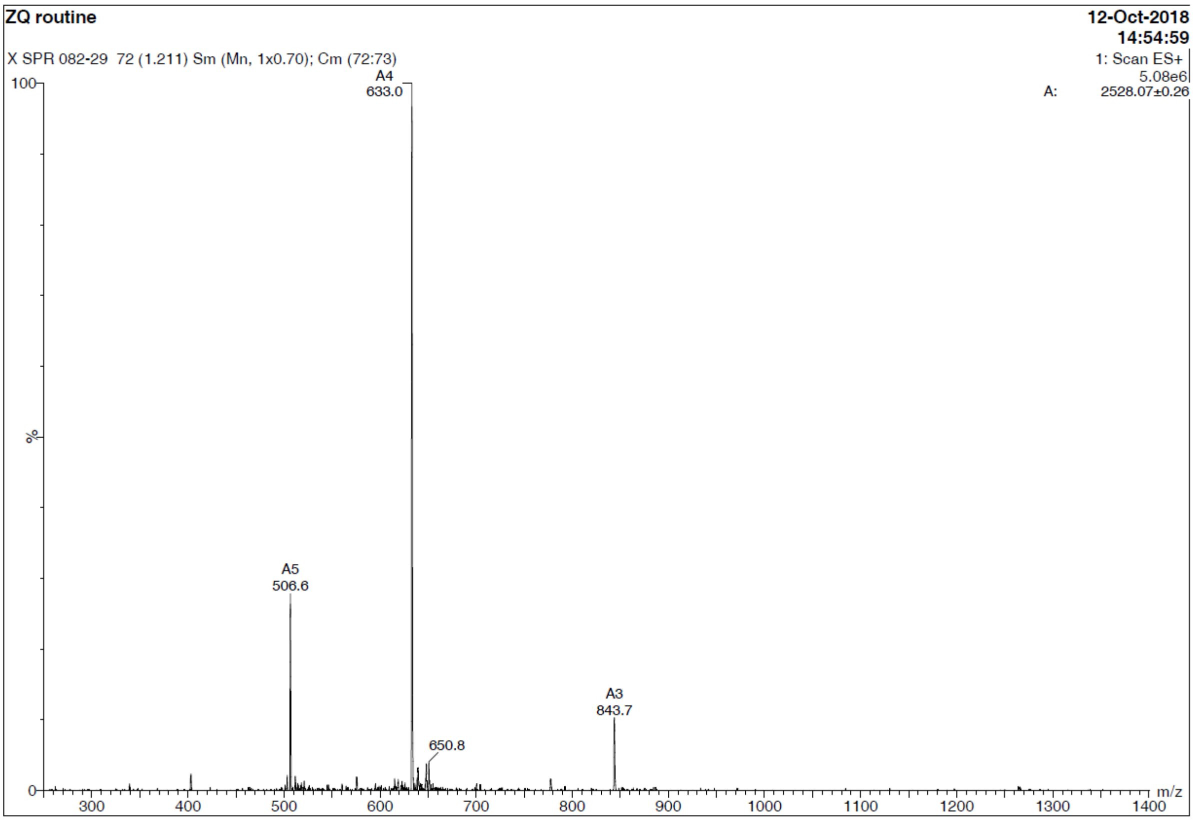

Figure S3. LC-MS profile of JMV 7110.

High Resolution Mass Result

**Analysis Info**		Acquisition Date	11/29/2019 2:54:06 PM	
Sample Name	**JMV 7110**	Instrument / Ser#	micrOTOF-Q	228888.10300

Acquisition Parameter

Source Type	ESI	Ion Polarity	Positive	Scan Begin	50 m/z	Scan End	2200 m/z

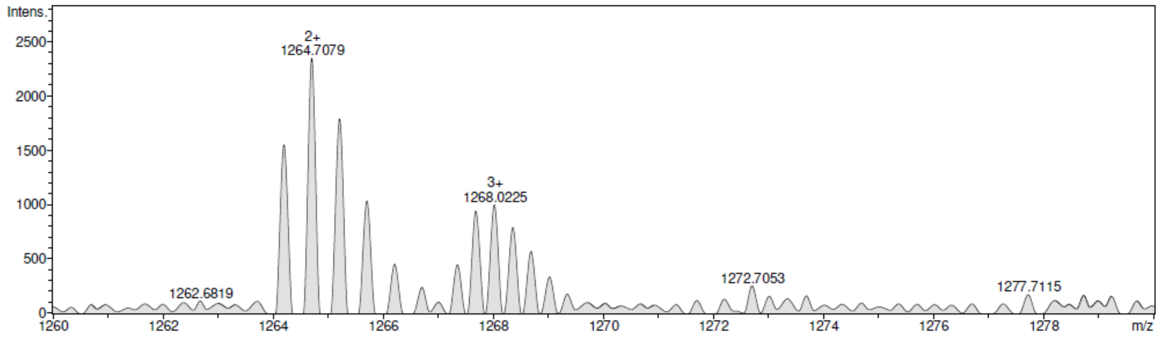

Meas. m/z	#	Ion Formula	Score	m/z	err [mDa]	err [ppm]	rdb	e⁻ Conf	N-Rule
1264.2069	1	$C_{123}H_{191}N_{26}O_{31}$	73.96	1264.2079	1.0	0.8	41.5	odd	ok
	2	$C_{122}H_{185}N_{33}O_{26}$	72.57	1264.2079	1.0	0.8	47.0	even	ok
➡	3	$C_{121}H_{189}N_{29}O_{30}$	100.00	1264.2072	-0.3	-0.2	42.0	even	ok
	4	$C_{120}H_{193}N_{25}O_{34}$	94.66	1264.2065	0.4	0.3	37.0	even	ok
	5	$C_{119}H_{187}N_{32}O_{29}$	92.72	1264.2065	0.4	0.3	42.5	odd	ok

Bruker Compass DataAnalysis 4.1 printed: 11/29/2019 3:21:21 PM Page 1 of 1

Figure S4. HRMS profile of JMV 7110.

3. Synthesis of linear heterodimer JMV 7253

JMV 7253 was synthesized followed the procedure above discussed for JMV 7110 (Scheme S2). In this case, 2-Cl-trytil choride resine was used. Fmoc-TMSAla-OH was loaded on the resin followed a protocol recently reported by us, and 0.8 mmol/g of loading was obtained.[2] Yield 5%. Purity 98%. ESI-MS (t_R 1.21 min): 853.5 [(M+3H$^+$)/3] 640.4 [(M+4H$^+$)/4], 512.5 [(M+5H$^+$)/5]. HPLC t_R 2.16 min; HRMS for $C_{121}H_{189}N_{29}O_{30}Si$ = calcd.: 1279.7054 [(M+2H$^+$)/2], 853.4729 [(M+3H$^+$)/3], and 640.3566 [(M+4H$^+$)/4].; found: 1279.7019 [(M+2H$^+$)/2], 853.4734 [(M+3H$^+$)/3], and 640.3571 [(M+4H$^+$)/4].

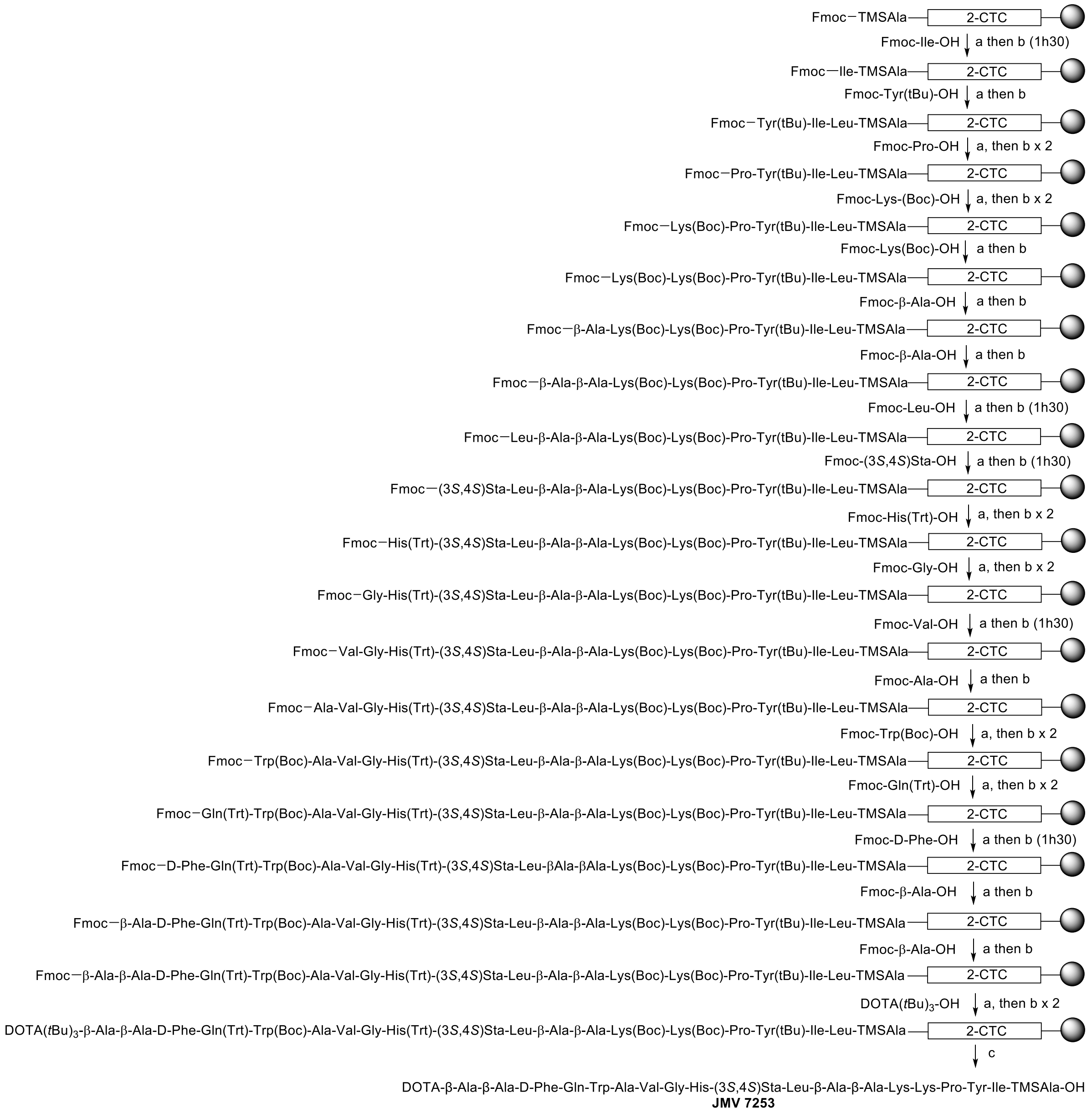

Figure S5. Reagents and conditions: a) 20% piperidine in DMF (5 min x 3), rt; b) Appropriate amino acid, HATU, DIPEA, DMF, 45 min, rt; c) TFA/DCM/TIS 90:5:5, 12h, rt. The shaking time and “x 2” reported in brackets refer exclusively to conditions “b”.

4. Characterization of JMV 7253

**Sample name:**	SPR117_prep_55
**Data file:**	C:\Chem32\LC1220_000\Agilent1220_000<br>\Data\SantoPreviti\SPR117_prep_552019-03-0616-10-28.D
**Description:**	
**Injection volume:**	5.000
**Instrument:**	LC 1220
**Injection date:**	3/6/2019 4:31:45 PM
**Acq. method:**	0a100_3min_214nm.M

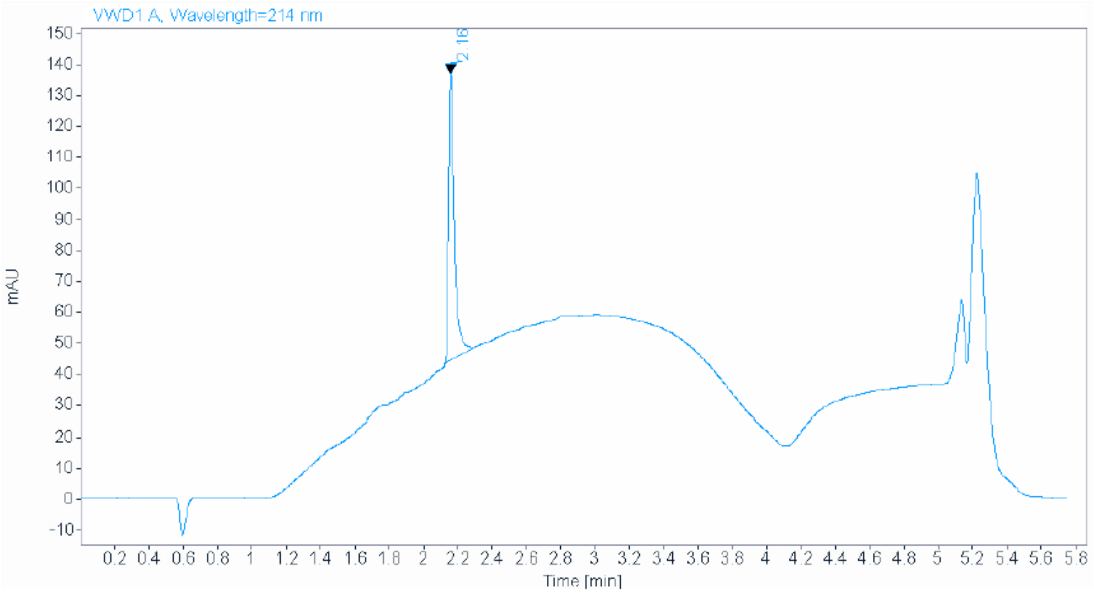

Signal: VWD1 A, Wavelength=214 nm

RT [min]	Area	Height	Peak Area Percent
2.160	216.76286	92.11746	100.00

Figure S6. HPLC profile of JMV 7253.

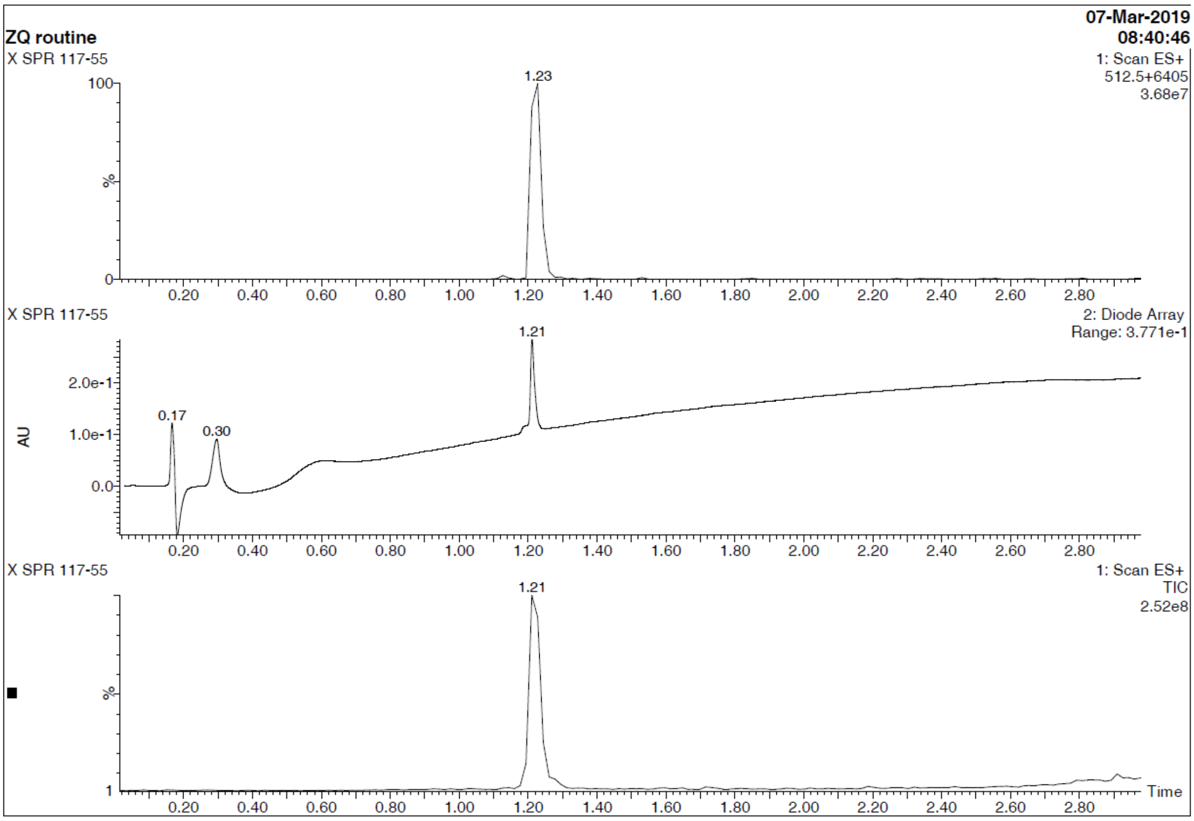

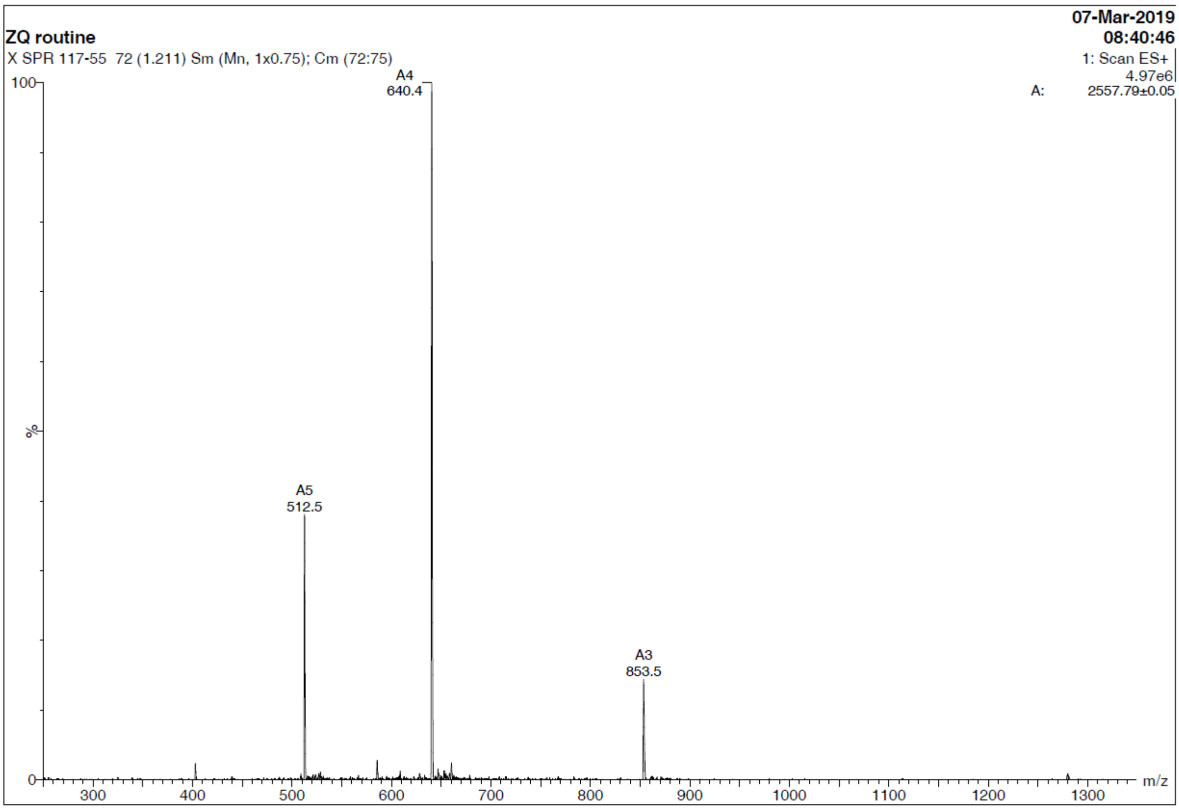

Figure S7. LC-MS profile of JMV 7253.

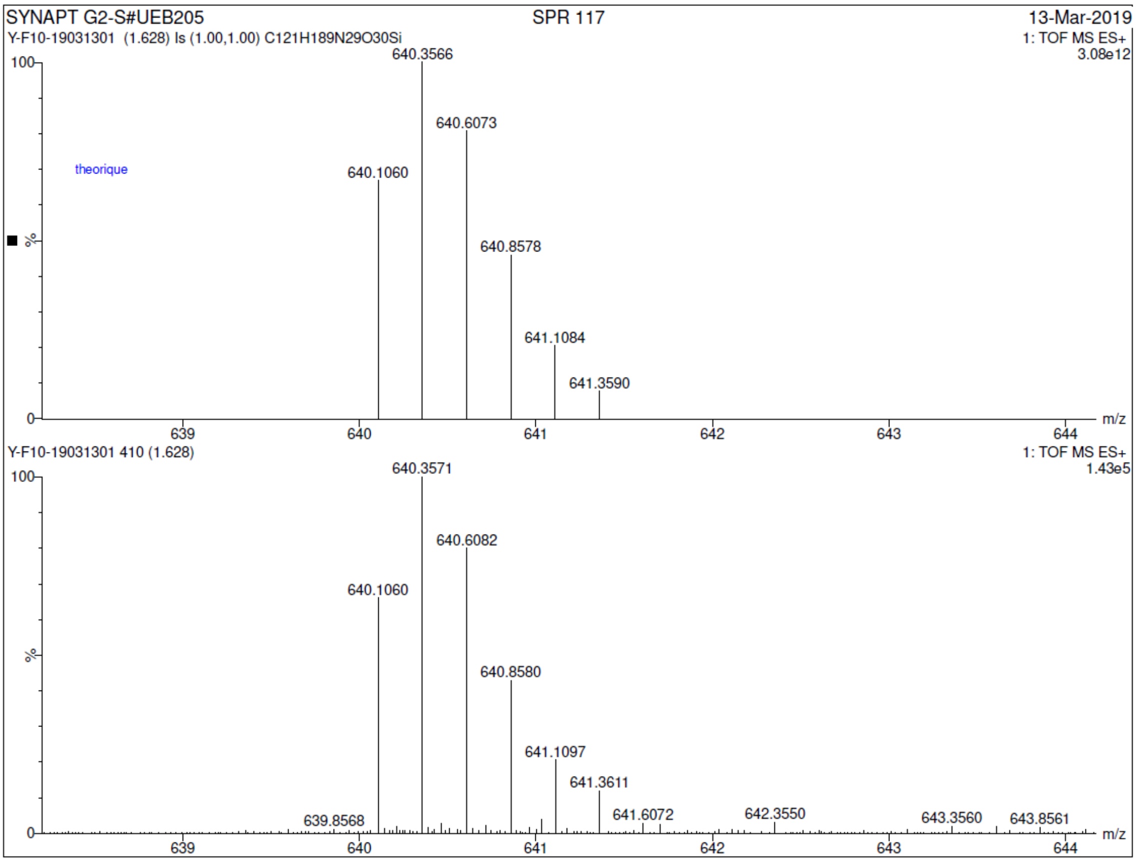

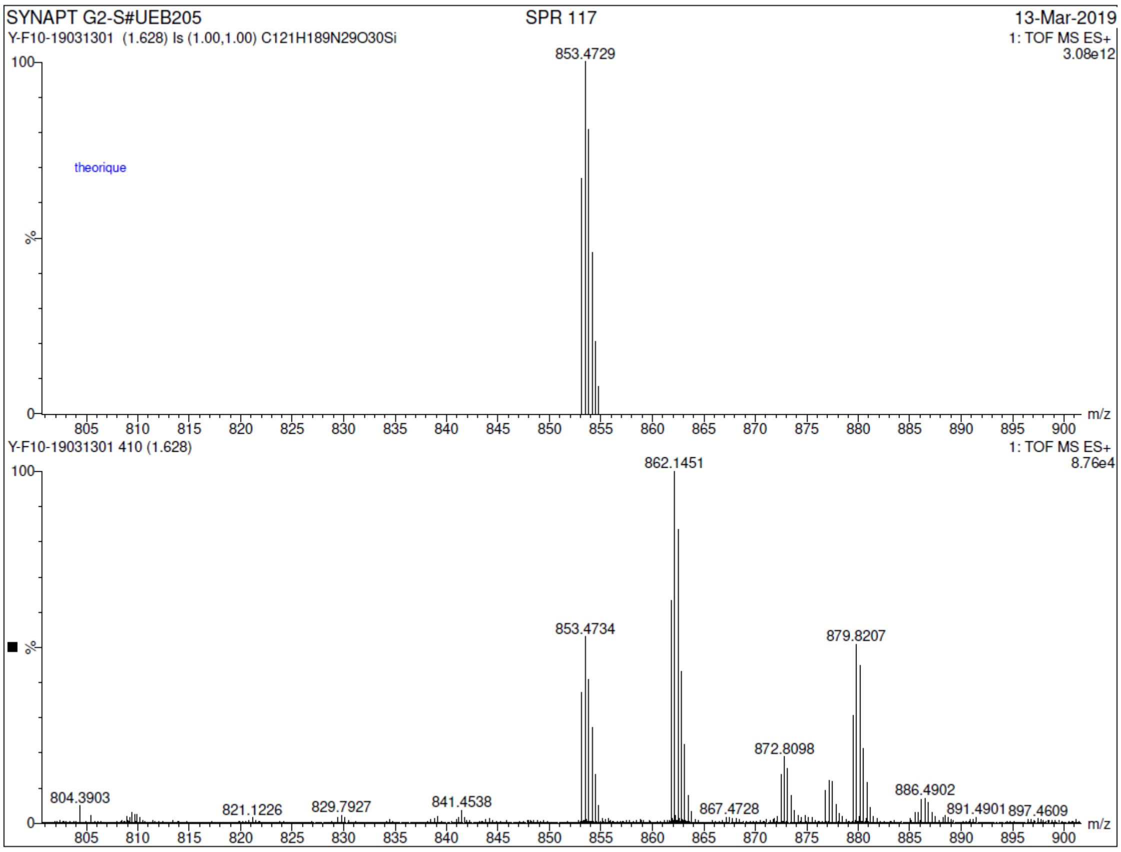

Figure S8. HRMS profile of JMV 7253.

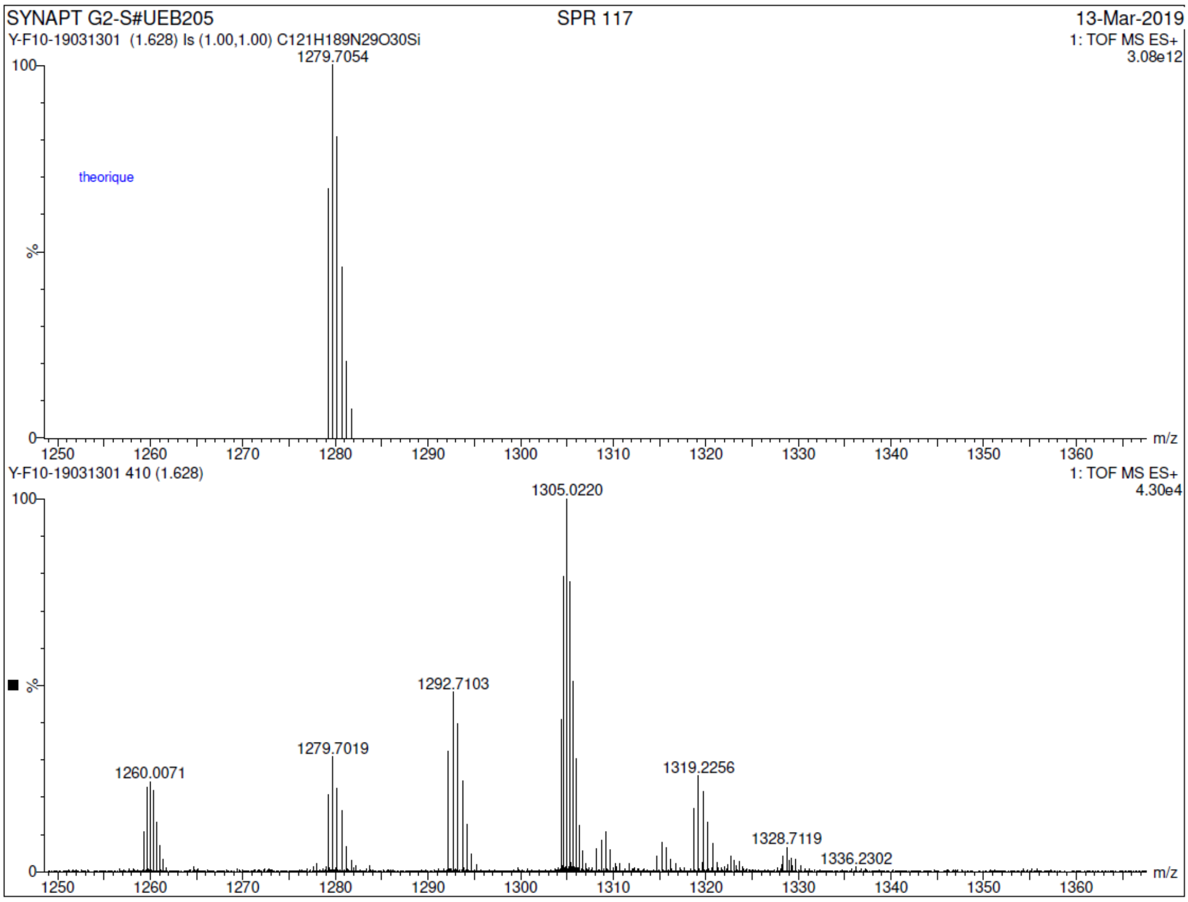

Figure S9. HRMS profile of JMV 7253 (continued).

5. Synthesis of branched heterodimer JMV 7266 and its intermediates

General procedure for coupling reactions in solution (GP1)

In a round bottom flask (A), the appropriate commercially available *N*-Fmoc-protected amino acid (1.5 eq.) was dissolved in DMF (10 mL/mmol) and HATU (1.5 eq.) and DIPEA (2 eq.) were added. The solution was maintained in stirring for 30 min. In a round bottom flask (B), the appropriate amine (1 eq.) was dissolved in DMF (10 mL/mmol), DIPEA (1.5 eq.) was added, and the pH was checked (> 7). The solution of the flask B was added to the flask A, pH was checked (~ 8), and the solution was maintained in vigorous stirring over night. Subsequently, DMF was removed *in vacuo* e the resulting residue was dissolved in EtOAc, washed with 1 M HCl (x 2), $NaHCO_3$ saturated solution (x 2), and brine (x 2), dried over $MgSO_4$, and concentrated *in vacuo*. The desired compound was purified by column chromatography using a EtOAc/cyclohexane as eluent mixture.

General procedure for Fmoc-deprotection in solution (GP2)

In a round bottom flask, the appropriate *N*-Fmoc-protected peptide (1 eq.) was dissolved in DMF (8 mL/mmol) and piperidine (2 mL/mmol) was added dropwise. The reaction was maintained in stirring for 1h. After this time, all volatiles were removed *in vacuo* and the obtained amine was used for the next step without purification.

Fmoc-Ile-Leu-OtBu (1)

The intermediate was synthesized following the general procedure GP1 (Scheme S3). In this reaction, Fmoc-Ile-OH and H-Leu-OtBu·HCl were used as the acid and the amine, respectively. Yield = 68%. Consistency = pale brown solid. ESI-MS (t_R 2.36 min): 523.3 (M + 1H), 467.2 (M – tBu), 245.2 (M – tBu – Fmoc).

H-Ile-Leu-OtBu (2)

Starting from Fmoc-Ile-Leu-OtBu **1**, the intermediate was obtained followed the general procedure GP2 (Scheme S3). Consistency = pale yellow oil. ESI-MS (t_R 1.26 min): 301.2 (M + 1H), 245.0 (M – tBu).

Fmoc-Tyr(tBu)-Ile-Leu-OtBu (3)

The intermediate was synthesized following the general procedure GP1 (Scheme S3). In this reaction, Fmoc-Tyr(tBu)-OH and H-Ile-Leu-OtBu **2** were used as the acid and amine, respectively. Yield = 75%. Consistency = pale orange solid. ESI-MS (t_R 2.60 min): 742.4 (M + 1H), 764 (M + 1Na), 686.4 (M + 1H – tBu), 555.3 (M – Leu-OtBu).

H-Tyr(tBu)-Ile-Leu-OtBu (4)

Starting from Fmoc-Tyr(tBu)-Ile-Leu-OtBu **3**, the intermediate was obtained followed the general procedure GP2 (Scheme S3). Consistency = pale yellow oil. ESI-MS (t_R 3.10 min): 520.7 (M + 1H), 542.7 (M + 1Na), 464.7 (M + 1H - tBu).

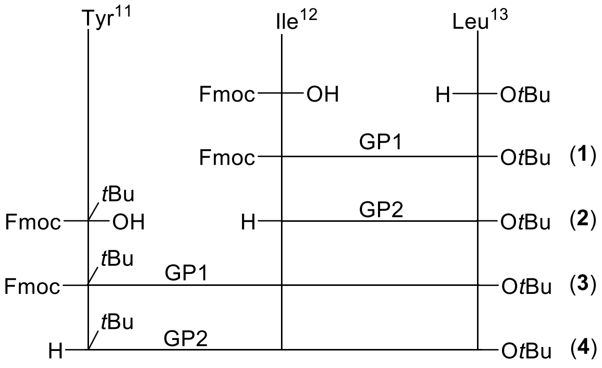

Figure S10. Reagents and conditions = GP1: HATU, DIPEA, DMF, rt, on; GP2: 20% piperidine in DMF, rt, 1h.

Synthesis of Fmoc-Glu(OMe)-βAla-Lys(Boc)-Lys(Boc)-Pro-OH (5)

The intermediate **5** was synthesized with SPPS, using 2-CTC as the resin in a scale of 1 mmol (Scheme S4). Fmoc-Pro-OH was loaded as mentioned above, and a loading of 0.68 mmol/g was obtained. A syringe of 20 mL was used. The subsequent amino acids were attached following the same procedure employed and fully described for JMV 7110, using 10 mL both of DMF and 20% piperidine in DMF for coupling reactions and Fmoc-deprotection, respectively. The cleavage was performed using a mixture of DCM/TFE/AcOH 8:1:1 (10 mL) in shaking for 12h. After that, volatiles were removed *in vacuo* and the obtained residue was used for the next step without purification. Consistency: colorless oil. ESI-MS (t_R 3.58 min): 1008.7 (M + 1H), 1030.6 (M + 1Na), 908 (M + 1H – Boc).

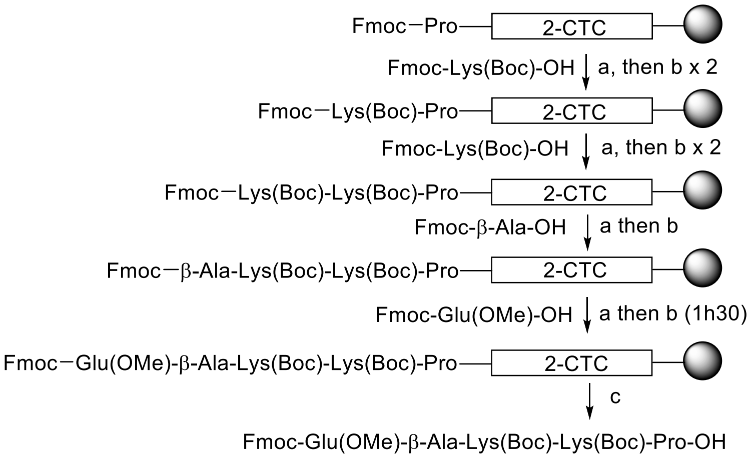

Figure S11. Reagents and conditions: a) 20% piperidine in DMF (5 min x 3), rt; b) Appropriate amino acid, HATU, DIPEA, DMF, 45 min, rt; c) DCM/TFE/AcOH 8:1:1, 12h, rt. The shaking time and "x 2" reported in brackets refer exclusively to conditions "b".

Synthesis of Fmoc-Glu(OMe)-βAla-Lys(Boc)-Lys(Boc)-Pro-Tyr(tBu)-Ile-Leu-OtBu (6)

The intermediate was synthesized following the general procedure GP1. In this reaction, Fmoc-Glu(OMe)-βAla-Lys(Boc)-Lys(Boc)-Pro-OH **5** and H-Tyr(*t*Bu)-Ile-Leu-O*t*Bu **4** were used as the acid and the amine, respectively. Yield = 31%. Consistency = pale orange solid. ESI-MS (t_R 2.54 min): 1510.6 (M + 1H), 1531 (M + 1Na).

Synthesis of Fmoc-Glu-βAla-Lys(Boc)-Lys(Boc)-Pro-Tyr(tBu)-Ile-Leu-OtBu (7)

In a round bottom flask, Fmoc-Glu(OMe)-βAla-Lys(Boc)-Lys(Boc)-Pro-Tyr(OtBu)-Ile-Leu-OtBu **6** (1 eq.) was dissolved in a mixture of iPrOH/H_2O 7:3, which contains $CaCl_2$ (0.8 M) at rt. NaOH (1.2 eq.) was added portion wise over 10 min and the reaction was maintained in stirring at rt for 7 h. After this time, no presence of starting material was detected. Subsequently, the reaction was neutralized with 1 M AcOH, and the volatiles were removed *in vacuo*. The resulting solid residue was dissolved in MeOH, and cold water was added dropwise to precipitate the desired product, which was filtered and washed with cold water. The desired product was recovered from the filter and used for the next step without further purification. Yield = 52%. Consistency = pale orange solid. ESI-MS (t_R 2.44 min): 1495.9 (M + 1H), 1497.0 (M + 2H), 1498.0 (M + 3H), 1517.9 (M + 1Na), 1519.0 (M + 1Na + 1H).

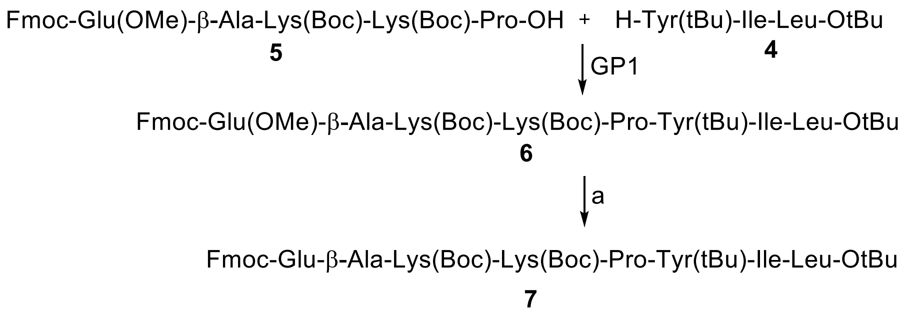

Figure S12. Reagents and condition = GP1: HATU, DIPEA, DMF, rt, on; a) NaOH, $CaCl_2$, *i*Pr-OH/H_2O (7:3), 7h, rt.

Synthesis of final compound JMV 7266

JMV 7266 was obtained using the SPPS approach starting from Fmoc-Leu Rink amide resin (0.4208 mmol/g, 0.08 mmol scale). Fmoc-deprotections and coupling reactions were performed using the same procedures above described for JMV 7110, as well as, the final cleavage (Scheme S6). Yield 2%. Purity 99%. ESI-MS (t_R 1.20 min): 886.2 [(M+3H$^+$)/3], 665.0 [(M+4H$^+$)/4]. HPLC t_R 2.12 min; HRMS for $C_{126}H_{195}N_{31}O_{32}$ = calcd.: 886.1616 [(M+3H$^+$)/3] and 664.8732 [(M+4H$^+$)/4]; found = 886.1642 [(M+3H$^+$)/3] and 664.8755 [(M+4H$^+$)/4].

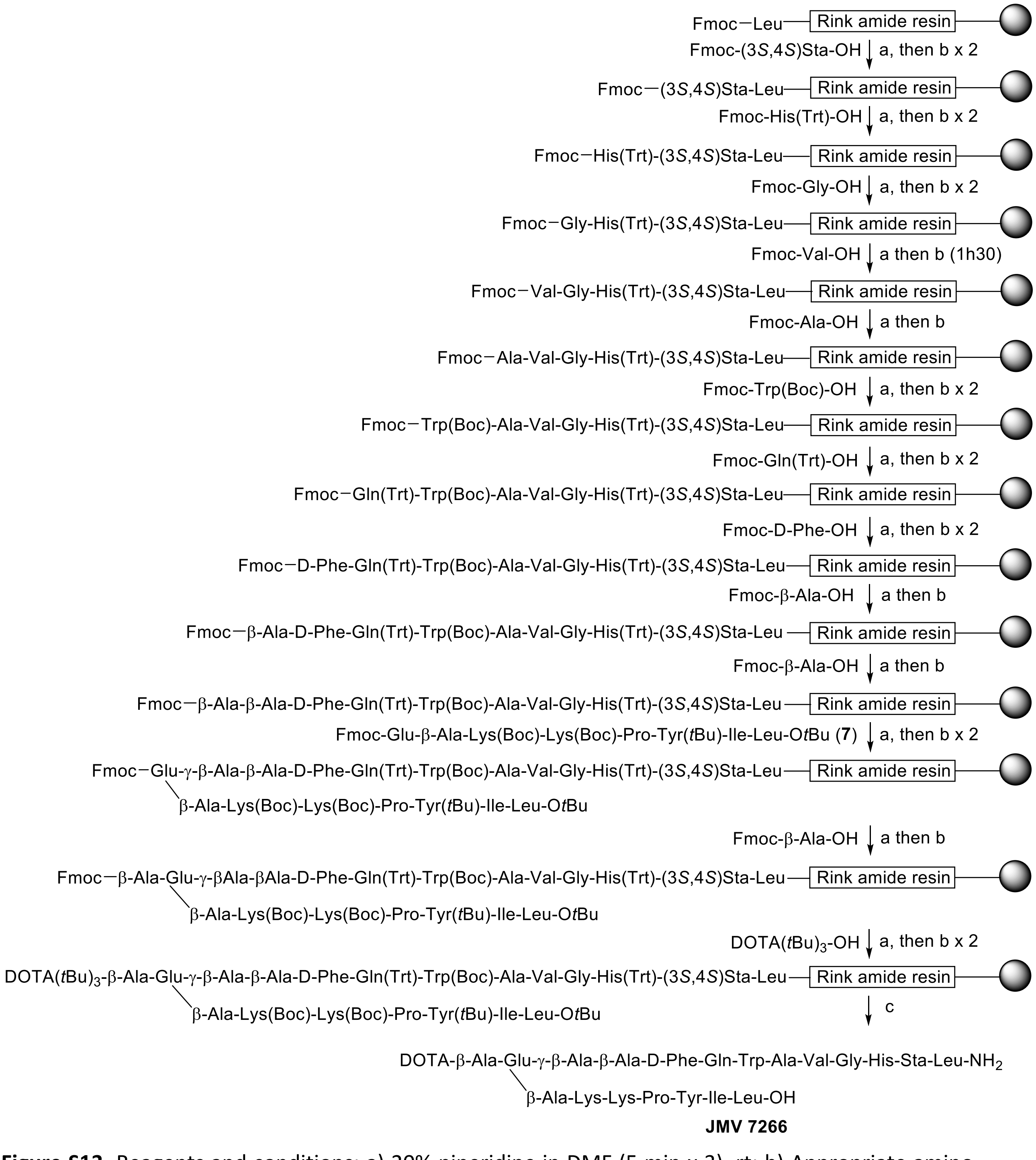

Figure S13. Reagents and conditions: a) 20% piperidine in DMF (5 min x 3), rt; b) Appropriate amino acid, HATU, DIPEA, DMF, 45 min, rt; c) TFA/DCM/TIS 90:5:5, 12h, rt. The shaking time and “x 2” reported in brackets refer exclusively to conditions “b”.

6. Characterization of JMV 7266

Sample name: SPR112_prep_66
Data file: C:\Chem32\LC1220_000\Agilent1220_000 \Data\SantoPreviti\SPR112_prep_662019-03-1912-40-27.D
Description:
Injection volume: 5.000

Instrument: LC 1220
Injection date: 3/19/2019 12:41:50 PM
Acq. method: 0a100_3min_214nm.M

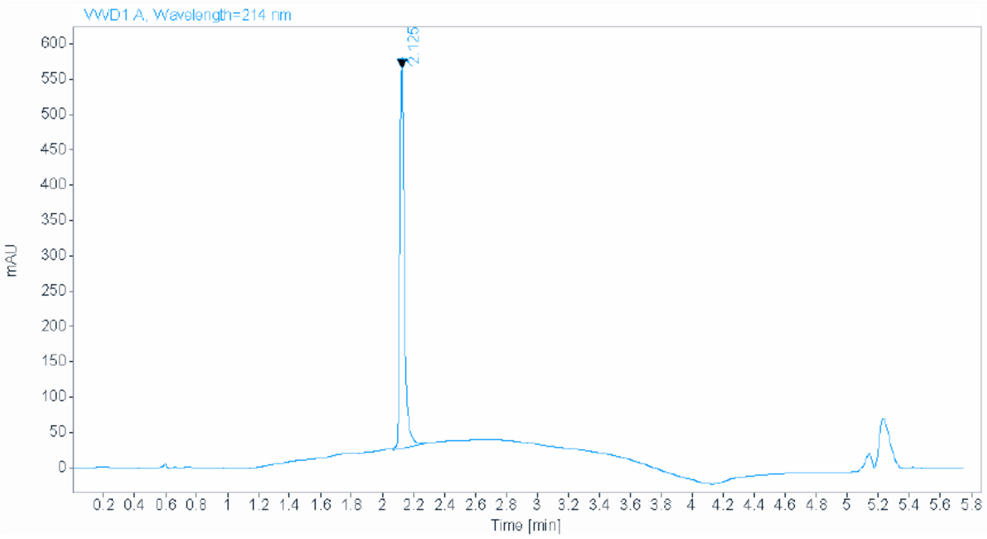

Signal: VWD1 A, Wavelength=214 nm

RT [min]	Area	Height	Peak Area Percent
2.125	1058.35083	538.28125	100.00

Figure S14. HPLC profile of JMV 7266.

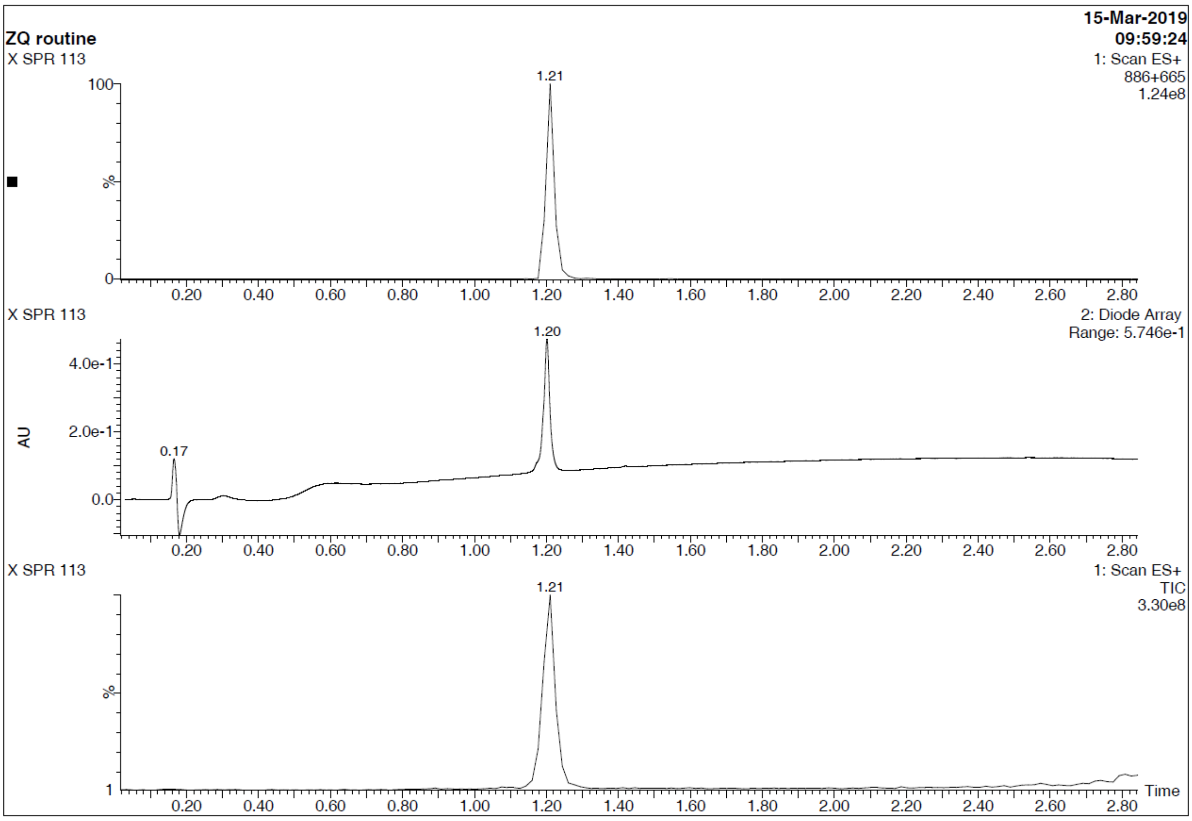

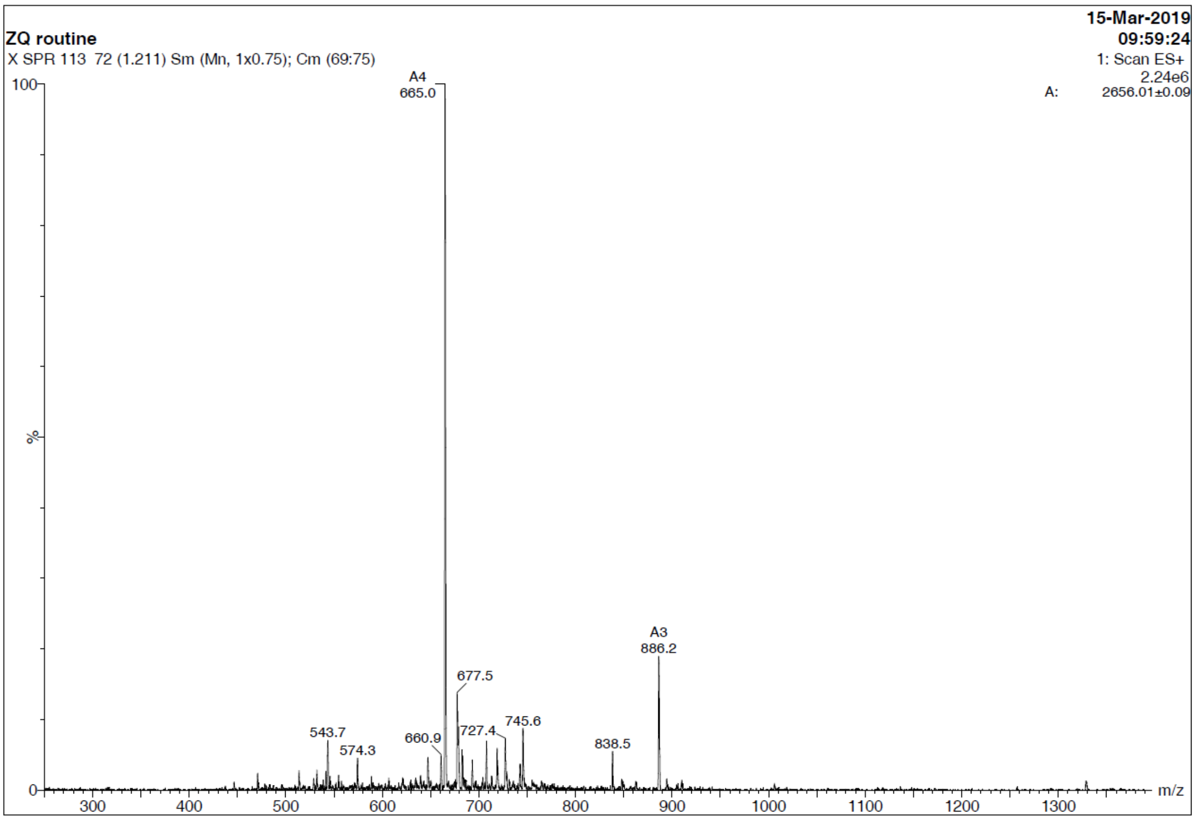

Figure S15. LC-MS of JMV 7266.

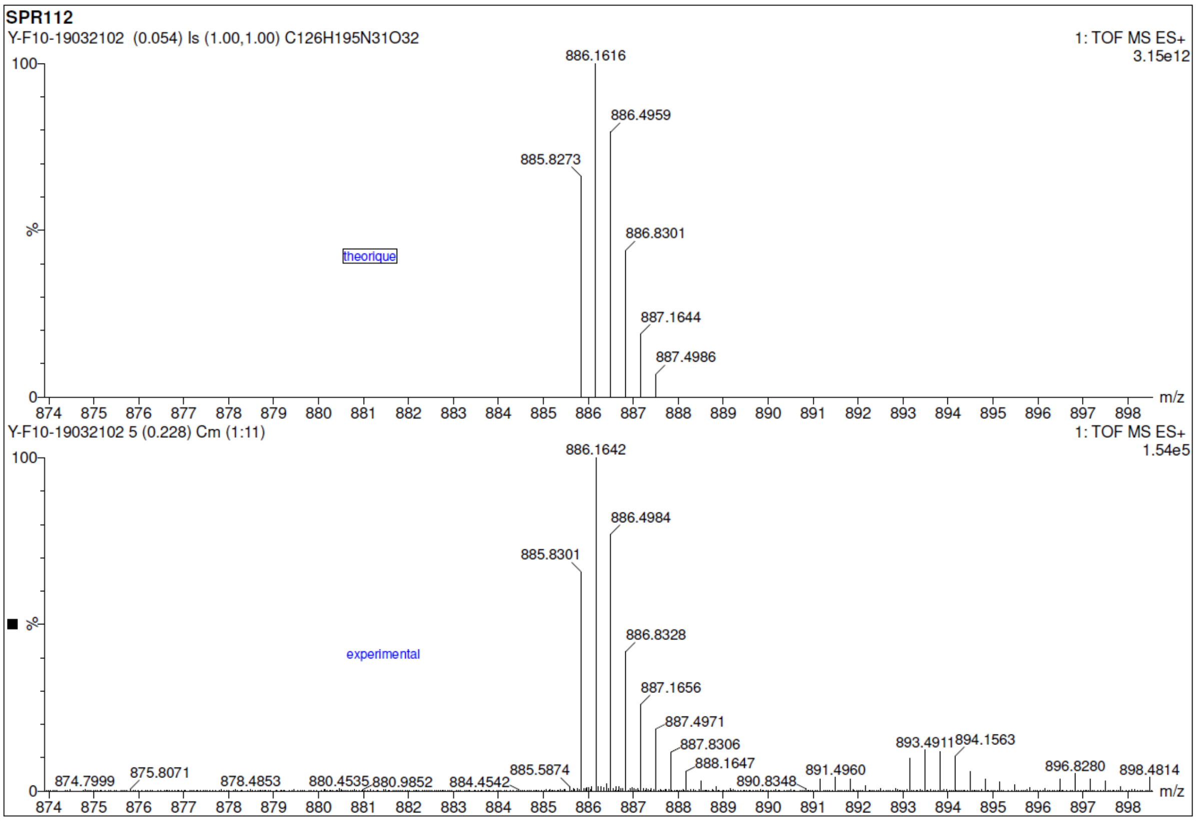

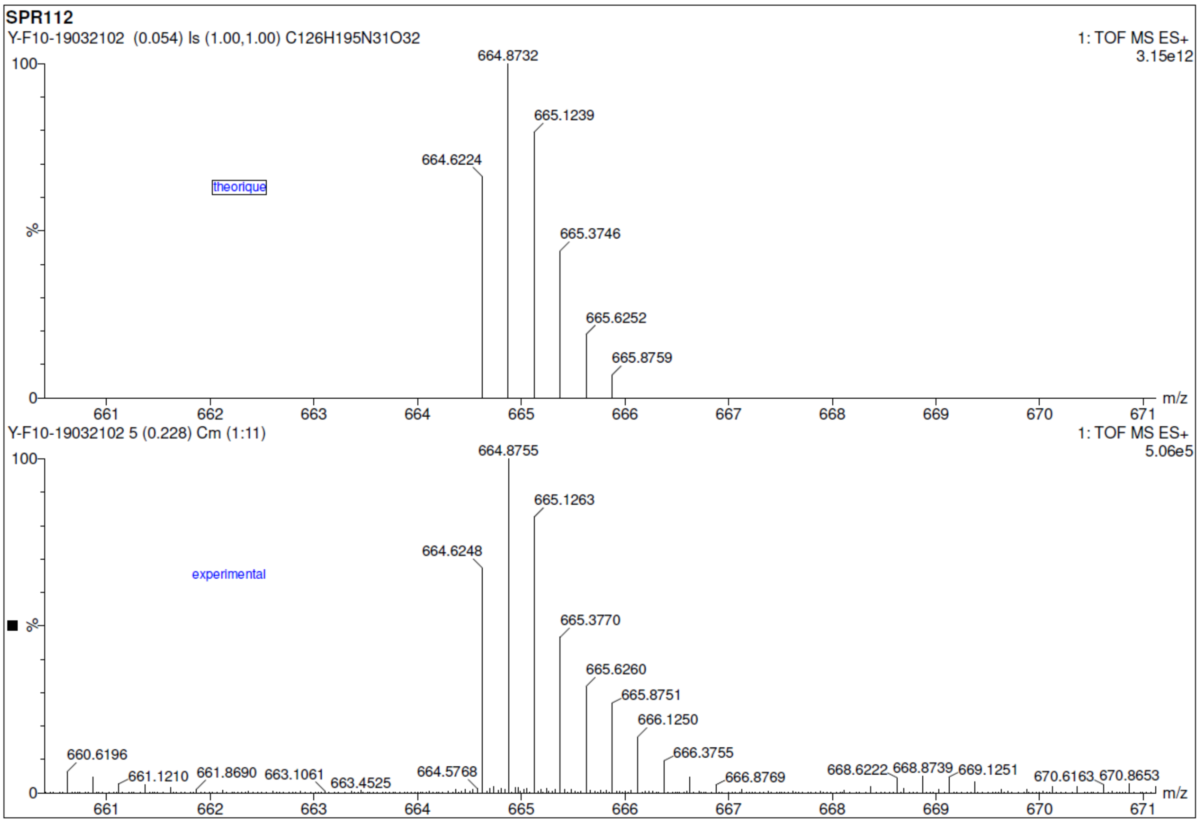

Figure S16. HRMS profile of JMV 7266

7. Radiolabeling of heterodimer compounds

a. [^{68}Ga]Ga-JMV 7110

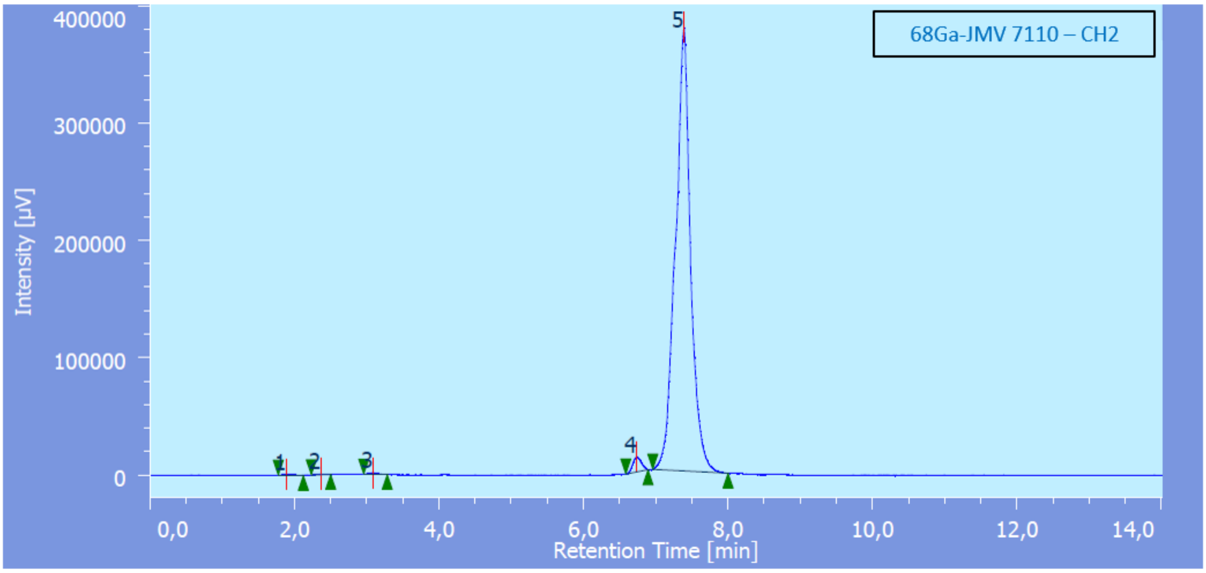

Figure S17. Radio-HPLC profile of [^{68}Ga]Ga-JMV 7110

b. [^{68}Ga]Ga-JMV 7253

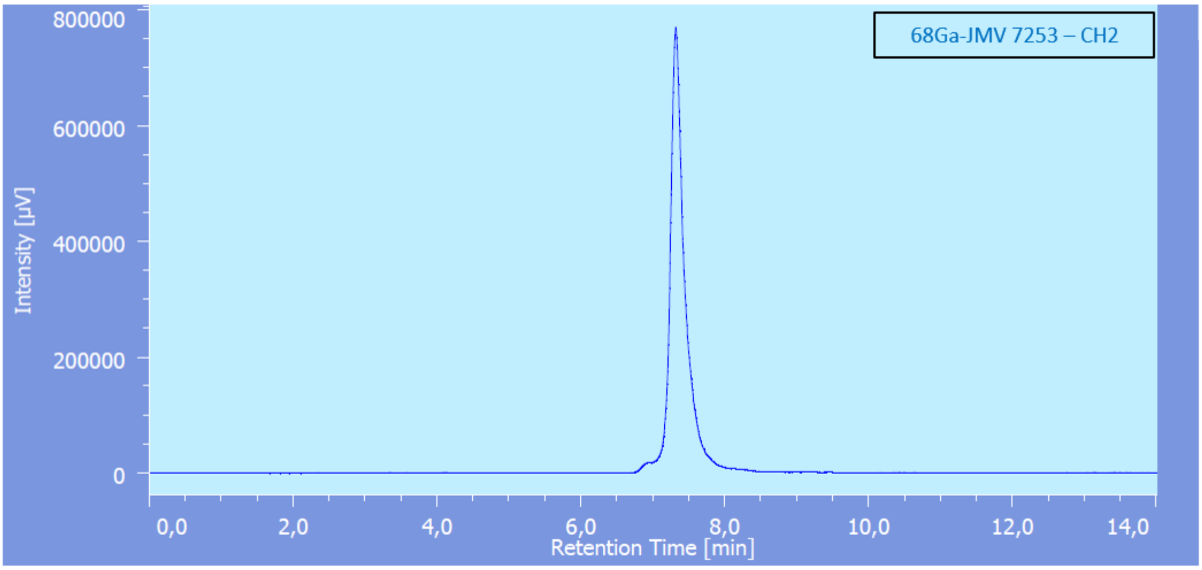

Figure S18. Radio-HPLC profile of [^{68}Ga]Ga-JMV 7253

c. [68Ga]Ga-JMV 7266

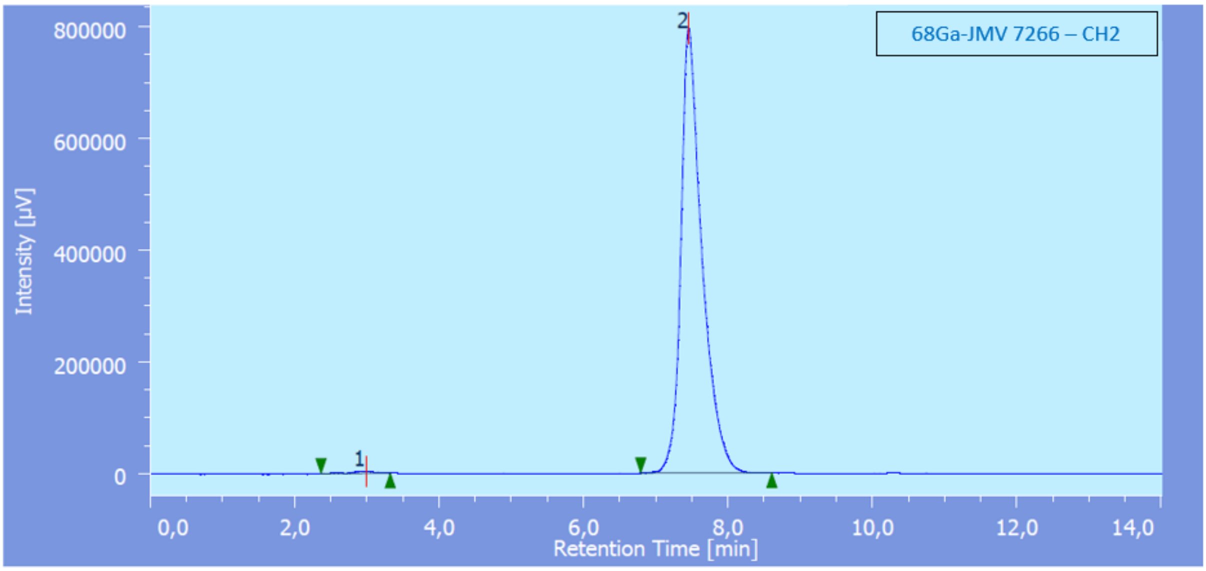

Figure S19. Radio-HPLC profile of [68Ga]Ga-JMV 7266
